# New approach toward the synthesis of deuterated pyrazolo[1,5-*a*]pyridines and 1,2,4-triazolo[1,5-*a*]pyridines

**DOI:** 10.3762/bjoc.13.80

**Published:** 2017-05-02

**Authors:** Aleksey Yu Vorob’ev, Vyacheslav I Supranovich, Gennady I Borodkin, Vyacheslav G Shubin

**Affiliations:** 1 Vorozhtsov Novosibirsk Institute of Organic Chemistry, Acad. Lavrentiev Ave. 9, Novosibirsk, 630090, Russia; 2 Novosibirsk State University, Pirogov st. 2, Novosibirsk, 630090, Russia

**Keywords:** deuteration, 1,3-dipolar cycloaddition, pyrazolo[1,5-*a*]pyridine, 1,2,4-triazolo[1,5-*a*]pyridine

## Abstract

An efficient and operationally simple synthesis of 7-deuteropyrazolo[1,5-*a*]pyridine and 7-deutero-1,2,4-triazolo[1,5-*a*]pyridine derivatives using α-H/D exchange of 1-aminopyridinium cations in basic D_2_O followed by a 1,3-cycloaddition of acetylenes and nitriles is presented. A high regioselectivity and a high degree of deuterium incorporation were achieved. The procedure was applied for several 4-R-1-aminopyridinium cations (R = H, Me, OMe).

## Introduction

Isotopically labeled compounds find broad applications in studies of chemical and biochemical reaction mechanisms and metabolism pathways. Deuterium is the most common used isotopic label in mechanistic studies. Deuterated organic compounds are widely used in biological [1] and pharmacological [2–5] investigations. In the last years deuteration became also an efficient tool in drug design [6].

Pyrazolo[1,5-*a*]pyridine and 1,2,4-triazolo[1,5-*a*]pyridine scaffolds attracted significant attention to the medicinal chemistry community during the past decade. For example, pyrazolo[1,5-*a*]pyridine derivatives were used in the design of antiviral [7–8], antimalarial [9] and antitubercular [10] agents. Also they were applied in the development of FIXa [11], PI3K [12], EGFR [13] and PDE [14] inhibitors and dopamine receptor ligands [15]. The nonselective PDE3,4 inhibitor ibudilast (MN-166) has been marketed in Japan for over 25 years for treating asthma and post-stroke patients [16]. 1,2,4-Triazolo[1,5-*a*]pyridines show antifungal [17], antitumor [18], and cytotoxic [19] activities. Both types of heterocyclic cores are readily available from *N*-aminopyridium salts and related pyridinium-*N*-imines via 1,3-cycloaddition reaction [20] or intramolecular ring closure [21–24]. The importance of these cores for medical chemistry studies suggests that isotopically labeled pyrazolo[1,5-*a*]pyridines and triazolo[1,5-*a*]pyridines could be of interest. Recently, deuterium-labeled pyridinium-*N*-imines were applied to mechanistic studies of the conversion to pyrazolo[1,5-*a*]pyridines [25–26]. Such labeled *N*-imines were obtained starting from commercially available pyridine-*d*_5_. Since substituted deuterated pyridines are less accessible new mild and simple methods of deuterium introduction into the pyridine ring are of great interest. The *N*-aminopyridinium cation has been shown to undergo a fast H/D exchange at the α-position of the pyridine ring [27]. Pyridine-*N*-imines could also be deuterated under significantly harder conditions [28–29]. In the present study we report mild and effective syntheses of 7-deuteropyrazolo[1,5-*a*]pyridine and 7-deutero-1,2,4-triazolo[1,5-*a*]pyridine derivatives by H/D exchange of 1-aminopyridinium cations followed by the reaction with acetylenes and nitriles.

## Results and Discussion

**Figure 1:**
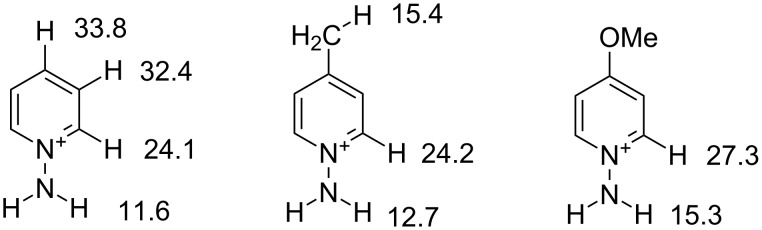
p*K*_a_ values for *N*-aminopyridinium cation hydrogen atoms according to DFT M06-2X 6-31+G(d,p) calculations.

*N*-Aminopyridinium salts are easily available via direct *N*-amination of parent pyridines. Salt **1a** was prepared by N-amination of pyridine with hydroxylamine-*O*-sulfonic acid followed by the reaction with HBF_4_ according to a previously described method [30]. Salts **1b,c** were prepared by direct N-amination of the corresponding pyridines with *O-*mesitylsulfonylhydroxylamine. In view of difficulties in obtaining experimental p*K*_a_ values of different positions of pyridinium cations we carried out DFT calculations [31] at the M06-2X 6-31+G(d,p) [32] level of theory with SMD [33] solvation (Figure 1, see also Supporting Information File 1).

**Scheme 1:**
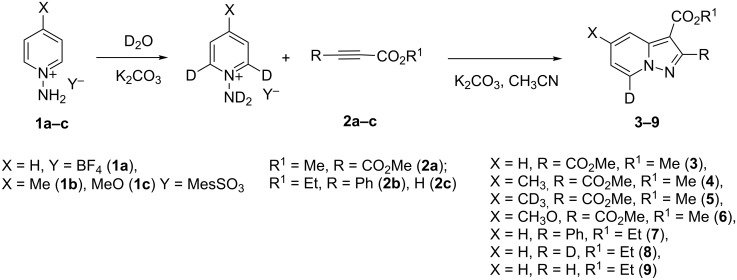
H/D exchange of *N*-aminopyridinium salts **1a–c** and their reaction with acetylenes.

As expected, in all cases the NH_2_ group is the most acidic. The NH_2_ group is usually ≈12–13 p*K*_a_ units more acidic than α-C–H hydrogens. However, the difference in p*K*_a_ of NH_2_ and CH_3_ groups of the 4-methyl-1-aminopyridinium cation is not so high and the NH_2_ group is only 2.7 units more acidic. 1-Aminopyridinium and 4-methyl-1-aminopyridinium cations have similar p*K*_a_ values for NH_2_ and α-C–H groups. The 4-methoxy-*N*-aminopyridinium cation possesses significantly lower acidity for both N–H and C–H hydrogens possibly due to the electron-donating effect of the methoxy group. These quantum chemical data together with Zoltewicz’s work [27] suggest 7-deuterium-labeled pyrazolo[1,5-*a*]pyridines could be obtained from *N*-aminopyridium salts through an H/D exchange in basic D_2_O solution followed by the cycloaddition reaction with acetylenes (Scheme 1).

In the first step, the H/D exchange in **1a** has been performed with a 0.67 M solution of K_2_CO_3_ in D_2_O at 80 °C for 5 min. After D_2_O evaporation and the reaction with dimethyl acetylenedicarboxylate (**2a**, DMAD) in MeCN D-labeled pyrazolo[1,5-*a*]pyridine **3** was obtained in 70% yield (Table 1). The deuteration at room temperature even for 24 h led to a significantly lower degree of deuteration (DD, 20%).

**Table 1:** Synthesis of deuteropyrazolo[1,5-*a*]pyridine derivatives^a^.

Product and D content (%)^b^	Yield (%)	Product and D content (%)^b^	Yield (%)

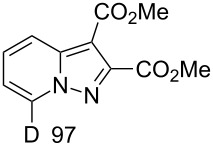 **3**	70	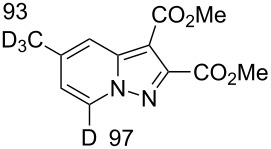 **5**	34^c^
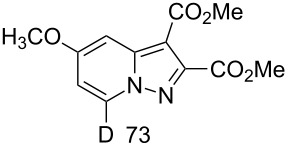 **6**	87	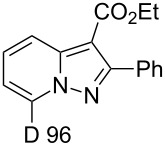 **7**	19^d^
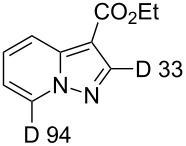 **8**, **9**	63		

^a^H/D-exchange: salt **1a–c** (0.20 mmol), K_2_CO_3_ (1.0 mmol), D_2_O (1.5 mL), 80 °C, 5 min; 1,3-cycloaddition: acetylene compound (0.2 mmol), MeCN (5 mL), chloranil (0.15 mmol), rt, 1 h. ^b^D content (%) was determined by ^1^H NMR. ^c^DD and yield after two runs in D_2_O. ^d^DD and yield after H/D exchange for 1 h in D_2_O at 80 °C.

Salt **1b** gave the corresponding 5-CD_3_-7-D-pyrazolopyridine **5** along with a 93% DD for the methyl group after two runs in D_2_O. The 4-methoxy derivative **1c** slowly underwent an H/D exchange at the 2-position of the pyridinium ring probably by reason of a lower acidity due to the electron-donating effect of the methoxy group. Thus, 5-methoxypyrazolopyridine **6** was obtained in 25% yield with a DD of only 58%. A higher DD of **6** could be achieved by increasing the reaction time, however, the yield of pyrazolopyridine decreased possibly due to the hydrolysis of the methoxy group. The 4-dimethylamino-substituted pyridinium-*N*-amine salt (anion MesSO_3_^−^) did not undergo an H/D exchange under the present conditions. Both 4-CO_2_Me-substituted pyridinium-*N*-amine and *N*-aminoisoquinolinium mesitylenesulfonates failed deuteration owing to the formation of insoluble compounds in basic D_2_O solution.

**Scheme 2:**
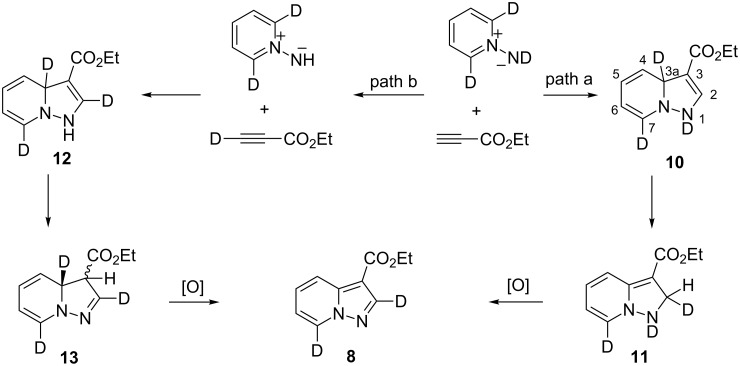
Possible pathways for the formation of **8**.

**Figure 2:**
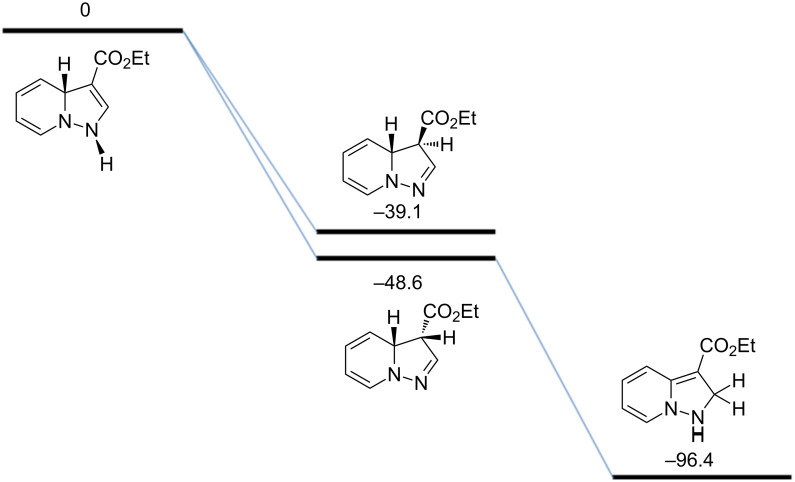
Relative stability of 3-CO_2_Et-substituted dihydropyrazolo[1,5-*a*]pyridines by the M06-2X 6-31+G(d,p) method in kJ/mol.

Ethyl phenylpropiolate (**2b**) reacts similar to DMAD with the formation of the corresponding 7-D-pyrazolopyridine **7**. When ethyl propiolate (**2c**) was used 2,7-dideuteropyrazolo[1,5-*a*]pyridine **8** was formed along with monodeuterated product **9**. The deuterium atom may appear in position 2 of compound **8** in two different ways (Scheme 2). The first one includes deuterium atom migration from position 3a to position 2 in cycloadduct **10** (path a) with the formation of intermediate **11** which, on further oxidation, gives product **8**. Intermediate **11** is the most stable isomer among other dihydro intermediates according to quantum chemistry calculations by the M-06-2X 6-31G+(d,p) method (Figure 2) and 3a -> 2 hydrogen atom migration is a highly exothermic process. However, the formation of **11** is probably kinetically unfavorable due to the prohibited 1,3-hydrogen shift. Thus, no NMR signals corresponding to **11** were found in the reaction mixture before oxidation. Another possible way of deuterium-atom incorporation includes the H/D exchange between the ND-group and the C_sp_–H hydrogen of ethyl propiolate before the formation of cycloadduct **12** (path b). Rearrangement of **12** into **13** and further oxidation leads to pyrazolopyridine **8**.

**Scheme 3:**
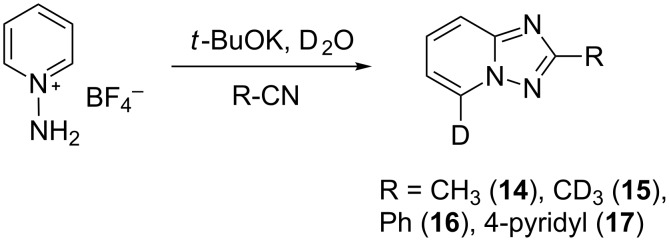
Synthesis of deutero 1,2,4-triazolo[1,5-*a*]pyridines.

In order to explore this approach for the synthesis of deutero-1,2,4-triazolo[1,5-*a*]pyridines the reaction of **1a** and MeCN in basic D_2_O solution was studied (Scheme 3, Table 2). The previously reported conditions for such a reaction were applied [18] except the fact that *t*-BuOK was used instead of KOH for KOD generation to achieve a higher degree of deuteration. The reaction yielded the corresponding 7-D-triazolo[1,5-*a*]pyridines with high D content at position 7. However, an H/D exchange at the methyl group was also observed with only moderate DD. The use of MeCN-*d*_3_ in place of MeCN resulted in the same yield of triazolopyridine-*d*_4 _**15**. Other nitriles such as PhCN and 4-cyanopyridine also gave the desired 7-D-triazolopyridines **16** and **17,** respectively.

**Table 2:** Synthesis of deutero 1,2,4-triazolo[1,5-*a*]pyridines^a^.

D content (%)^b^	Yield (%)	D content (%)^b^	Yield (%)

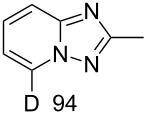 **14**	40	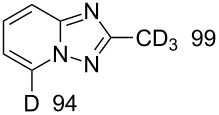 **15**	40
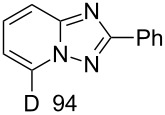 **16**	42	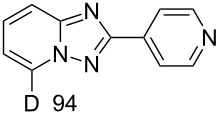 **17**	46

^a^Conditions: salt **1a** (0.20 mmol), RCN (0.2 mmol), *t*-BuOK (1.0 mmol), D_2_O (1.5 mL), rt, overnight under air. ^b^D content (%) was determined by ^1^H NMR.

## Conclusion

We have developed an efficient protocol for the synthesis of deuterium labeled pyrazolo[1,5-*a*]pyridines and triazolo[1,5-*a*]pyridines. Readily available and cheap D_2_O was employed as the deuterium source. The established system displays notable efficacy under mild reaction conditions in a short reaction time. A comparative assessment of the p*K*_a_ values of different positions of *N*-aminopyridinium cations by DFT calculations allows predicting the direction of deuterium exchange. We assume that this method could also be extended to tritium labelling of pharmaceutically interesting compounds for medicinal applications.

## Supporting Information

File 1 Experimental part, NMR spectra, and quantum calculation details.
