# Supplementary material for: New approach toward the synthesis of deuterated pyrazolo[1,5-a]pyridines and 1,2,4-triazolo[1,5-a]pyridines
Source: Beilstein J Org Chem. 2017 May 2;13:800–5. doi: 10.3762/bjoc.13.80 (PMC5433178; doi:10.3762/bjoc.13.80)
Supplement: File 1 — Experimental part, NMR spectra, and quantum calculation details. [file Beilstein_J_Org_Chem-13-800-s001.pdf]

Supporting information

for

New approach toward the synthesis of deuterated pyrazolo[1,5-*a*]pyridines and 1,2,4-triazolo[1,5-*a*]pyridines

Aleksey Yu. Vorob'ev[1,2*], Vyacheslav I. Supranovich[1,2], Gennady I. Borodkin[1,2], Vyacheslav G. Shubin[1]

Address: [1]Vorozhtsov Novosibirsk Institute of Organic Chemistry, Acad. Lavrentiev Ave. 9, Novosibirsk, 630090, Russia and [2]Novosibirsk State University, Pirogov st. 2, Novosibirsk, 630090, Russia

Email - Aleksey Yu. Vorob'ev - vor@nioch.nsc.ru

[*] Corresponding author

Experimental part, NMR spectra, and quantum calculation details

Table of content

**General information**	**S2**
**Synthesis of 7-Deuteropyrazolo[1,5-a]pyridines**	**S3**
**Synthesis of 7-deutero-1,2,4-triazolo[1,5-a]pyridines**	**S10**
**Quantum chemistry studies**	**S16**

General information

Chloranil was purified by sublimation. K_2CO_3 was dried at 400 °C. O-(Mesitylenesulfonyl)hydroxylamine (MSH) was obtained as described in the literature[1]. 1-Amino-4-X-pyridinium (X = CH_3, OMe, CO_2Me, NMe_2) and isoquinolinium mesitylenesulfonates were obtained as previously described[1]. Other starting materials were obtained from commercial supplies and used without purification unless otherwise specified.

The TLC was carried out on Sorbfil silica plates (UV 254). Visualization of the developed chromatograms was performed by UV light. Spectral and analytical studies were provided at Chemical Service Centre of Siberian Branch of the Russian Academy of Sciences. NMR spectra were recorded on a Bruker Avance-300 (300.13 MHz for ^{1}H) and Avance-400 (400.13 MHz for ^{1}H and 100.62 MHz for ^{13}C) spectrometers, using the residual proton and carbon signals of $(CD_3)_2SO$ (δ_H 2.50 ppm; δ_C 39.5 ppm) or $CDCl_3$ (δ_H 7.26 ppm; δ_C 77.0 ppm) as internal standards. ^{13}C NMR spectra were measured with C–H spin decoupling. Masses of molecular ions were determined by HRMS on a DFS Thermo scientific instrument (EI, 70 eV).

Incorporation of deuterium into heterocyclic moiety was confirmed by lowering the intensity of the corresponding signals in ^{1}H and ^{13}C NMR spectra.

N-Aminopyridinium tetrafluoroborate

N-Aminopyridinium tetrafluoroborate was obtained according to previously described procedure [2].

To a solution of 22.6 g. (0.20 mol) of hydroxylamine-O-sulfonic acid in 100 mL of water 48 mL (48 g, 0.60 mol) of pyridine was added. The resulted mixture is heated at about 90 °C for half an hour. After cooling to room temperature 27.6 g (0.20 mol) of potassium carbonate were added. The water and excess pyridine were removed under reduced pressure. The solid residue was extracted with 200 mL of ethanol. To the purple ethanol extract 40% aqueous HBF_4 was added dropwise until the purple color disappeared. The resulting solution was stored at −20 °C overnight. The solid that precipitated was collected by filtration and recrystalized from ethanol to give 25.8 g (70%) of 1-aminopyridinium tetrafluoroborate as pale yellow crystals. M.p. 142-143 ^{o}C. ^{1}H-NMR (DMSO-d_6): 8.00 (t, 2H, J = 7.8 Hz), 8.26 (t, 1H, J = 7.8 Hz), 8.45 (br s, 2H), 8.75 (d, 2H, J = 7.8 Hz).

Synthesis of 7-Deuteropyrazolo[1,5-*a*]pyridines

General procedure for 7-D pyrazolo[1,5-*a*]pyridines

N-Aminopyridinium salt (0.20 mmol) and K_2CO_3 (138mg, 1.00 mmol) in D_2O (0.83 mol, 1.5 mL) were heated for 5 min in water bath (80 °C). After cooling the mixture was evaporated under reduced pressure. Acetylene derivatives (0.20 mmol) in acetonitrile (5 mL) were added to the residue. The mixture was stirred for 10 min and then chloranile (0.20 mmol) was added. After an hour the mixture was evaporated, the residue rinsed with chloroform and the resulting solution was passed through a thin silica gel layer. Evaporation gave the product.

Dimethyl 7-D-pyrazolo[1,5-*a*]pyridine-2,3-dicarboxylate (3). [*cf.* Ref. [3]]

^{1}H-NMR ($CDCl_3$) 8.15 (dd, 1H, J = 1.4, 9.0 Hz), 7.44 (dd, 1H, J = 6.9, 9.0 Hz), 7.02 (dd, 1H, J = 1.2, 6.8 Hz), 4.01 (s, 3H), 3.91 (s, 3H) ppm. ^{13}C NMR ($CDCl_3$): 163.2, 162.6, 147.2, 141.4, 127.9, 119.8, 114.9, 102.7, 52.9, 51.7 ppm. HRMS (EI): M^+, found 235.0697. $C_{11}H_9DN_2O_4$ requires 235.0698.

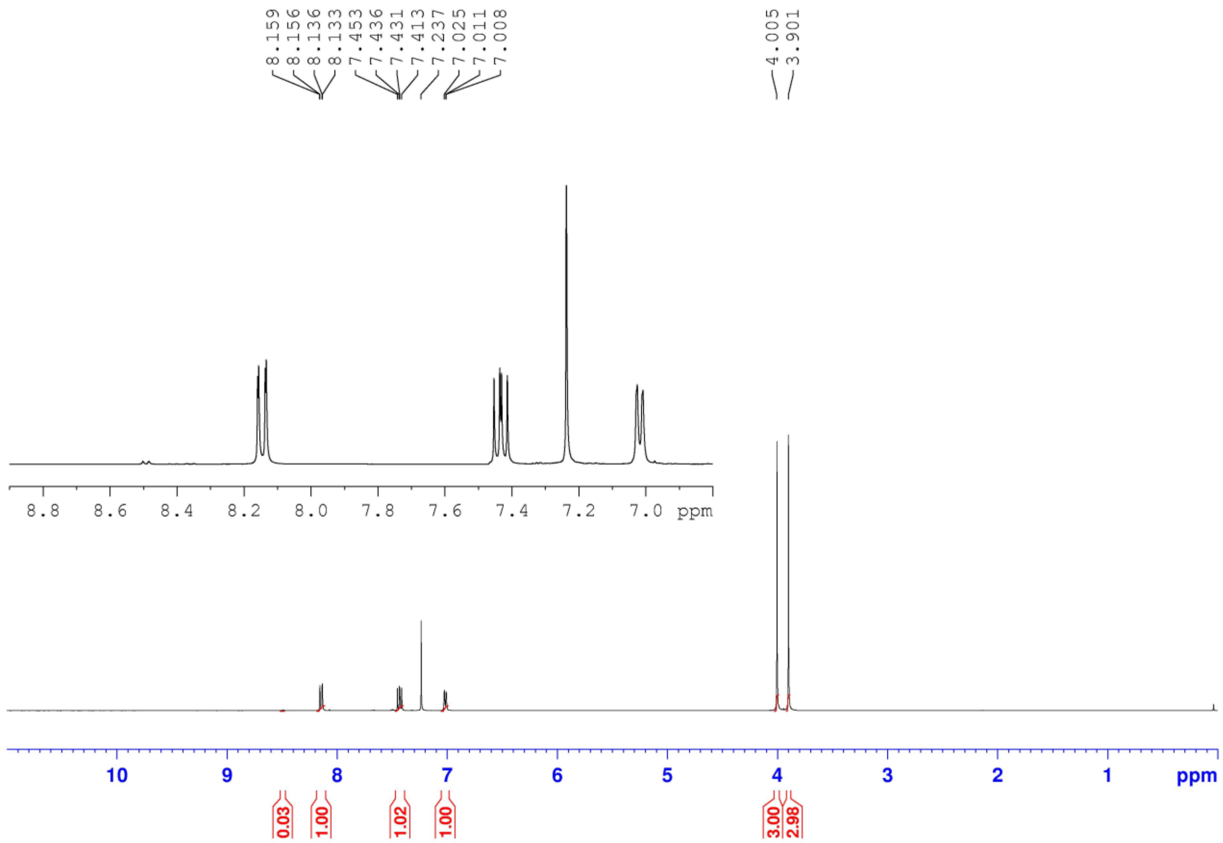

Figure S1. ^{1}H NMR of compound **3**.

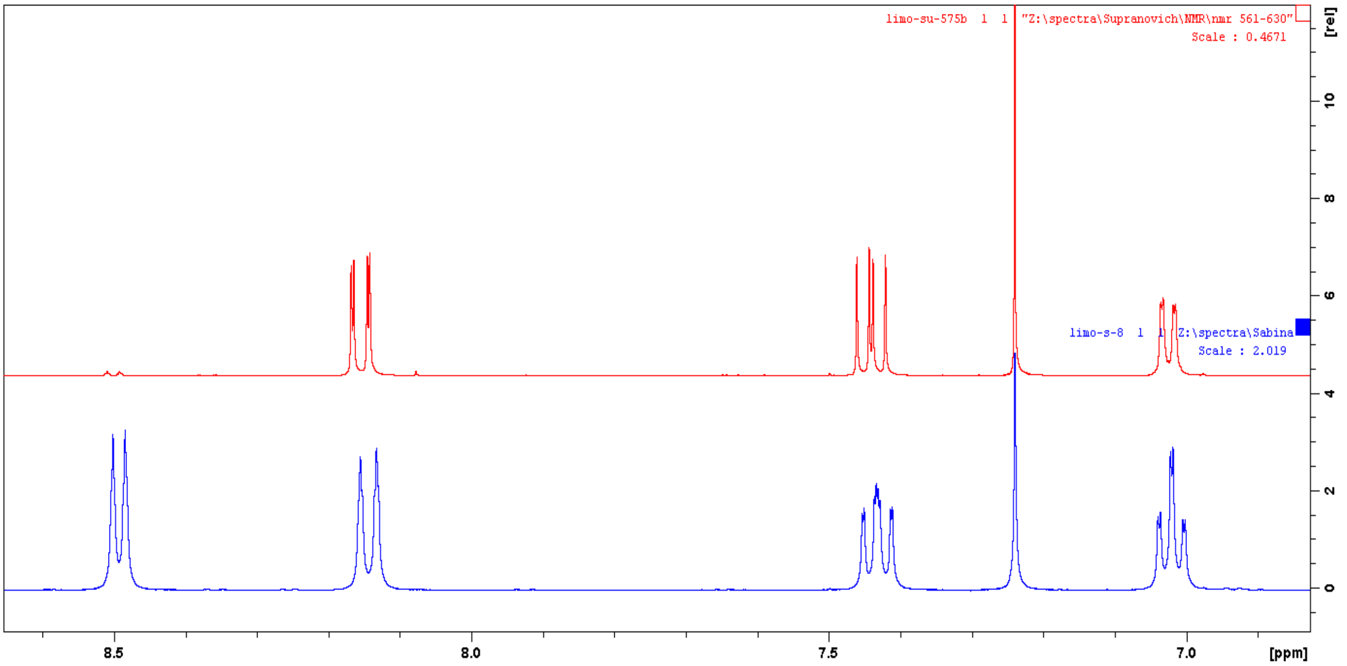

Figure S2. Comparision 1H NMR of **3** and nondeuterated dimethyl pyrazolo[1,5-*a*]pyridine-2,3-dicarboxylate.

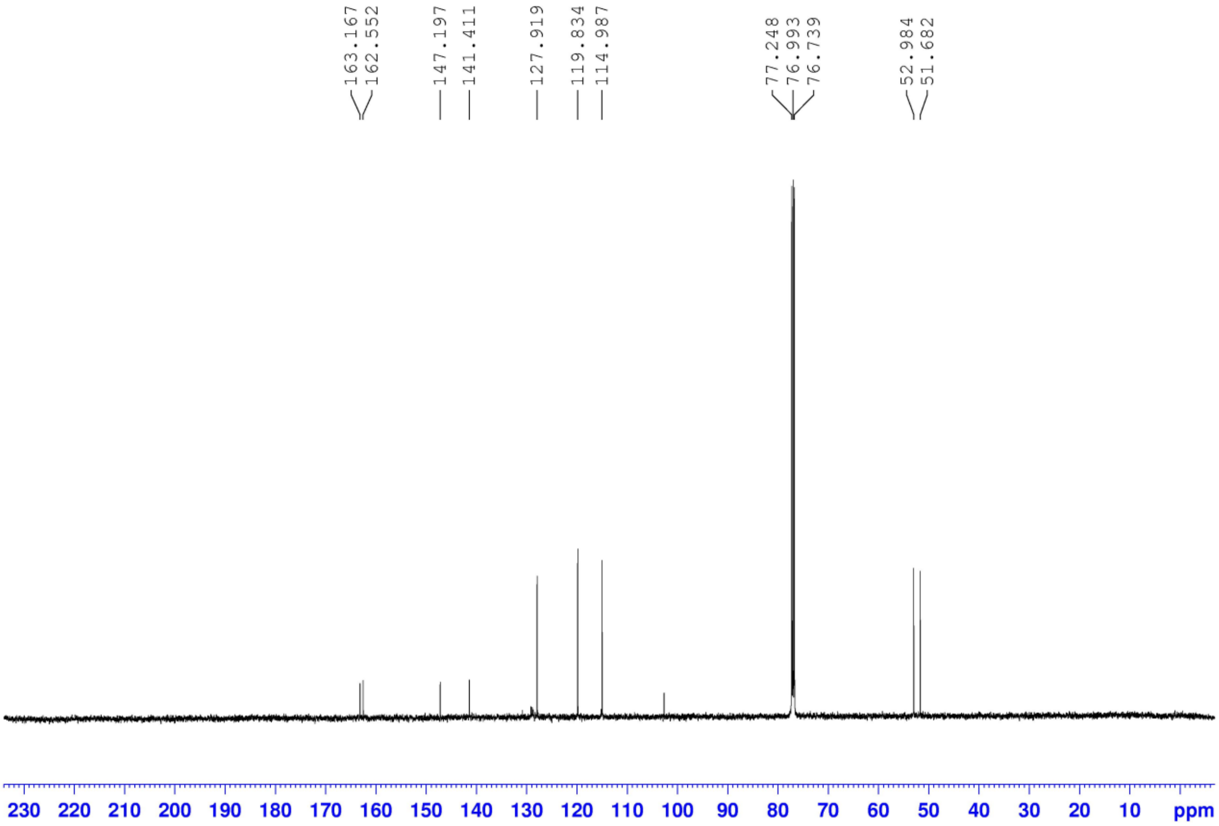

Figure S3. ^{13}C NMR of compound **3**.

Ethyl 2-phenyl-7-D-pyrazolo[1,5-*a*]pyridine-3-carboxylate (9). [*cf.* Ref.[4]]

^{1}H-NMR ($CDCl_3$): 8.19 (dd, 1H, J = 1.3, 9.0 Hz), 7.79-7.75 (m, 2H), 7.46-7.41 (m, 3H), 7.38 (dd, 1H, J = 6.9, 9.0 Hz), 6.92 (dd, 1H, J = 1.3, 7.0 Hz), 4.30 (q, 2H, J = 7.2 Hz), 1.29 (t, 3H, J = 7.2 Hz) ppm. ^{13}C NMR ($CDCl_3$): 163.6, 157.0, 142.7, 132.6, 130.0, 128.9, 127.8, 127.3, 119.8, 113.7, 100.8, 59.9, 14.3 ppm. HRMS (EI): M^+, found 267.1113. $C_{16}H_{13}DN_2O_2$ requires 267.1113.

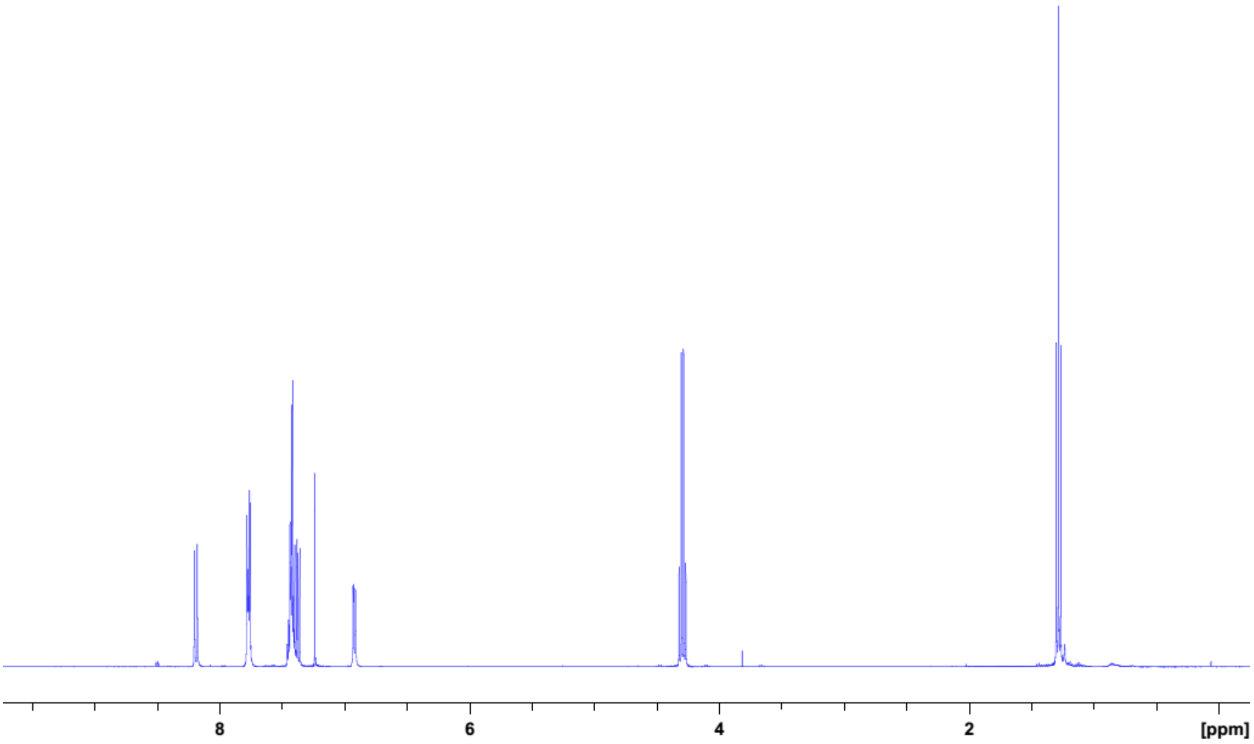

Figure S4. ^{1}H NMR of compound **9**.

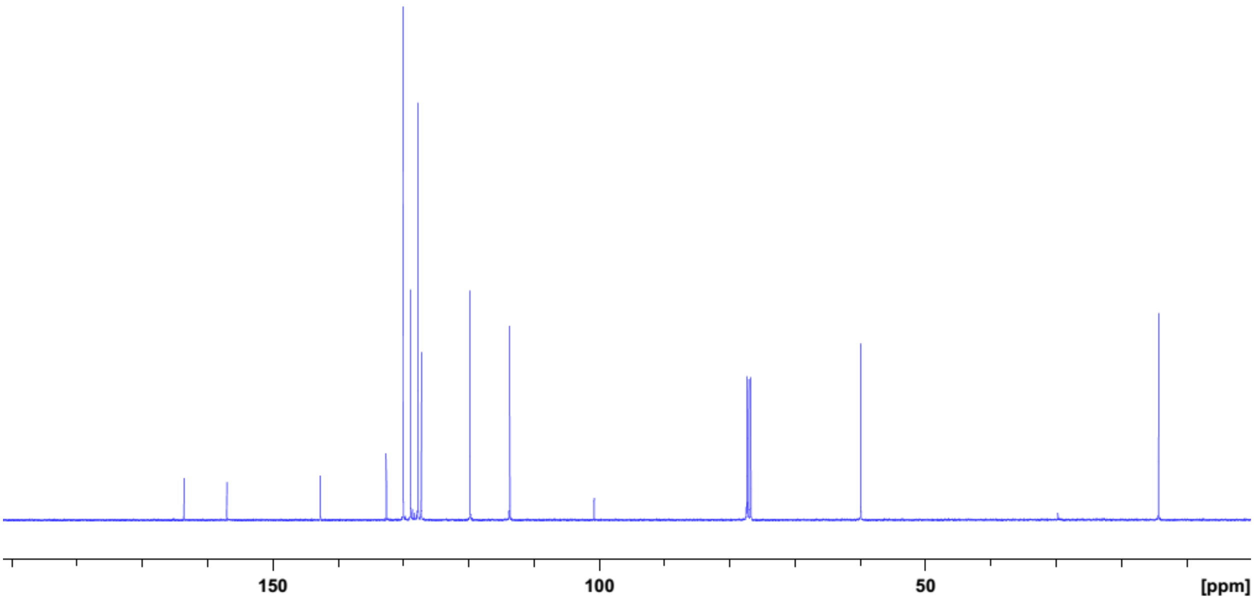

Figure S5. ^{13}C NMR of compound **9**.

Ethyl 7-D-pyrazolo[1,5-*a*]pyridine-3-carboxylate (mixture of **7, 8**). [*cf.* Ref.[5]]

24 mg (63%). ^{1}H-NMR ($CDCl_3$): 8.39 (s, 1H), 8.14 (dd, 1H, J =1.4, 8.9 Hz), 7.39 (dd, 1H, J = 6.9, 8.9 Hz), 6.93 (dd, 1H, J = 0.9, 6.9 Hz), 4.37 (quart, 2H, J = 7.2 Hz), 1.40 (t, 3H, J = 7.2 Hz) ppm. ^{13}C NMR ($CDCl_3$): 163.3, 144.7, 140.7, 127.1, 128.9 (t), 118.9, 113.5, 103.8, 113.3, 59.8, 14.4 ppm. HRMS (EI): M^+, found 191.0801. $C_{10}H_{19}DN_2O_2$ requires 191.0800.

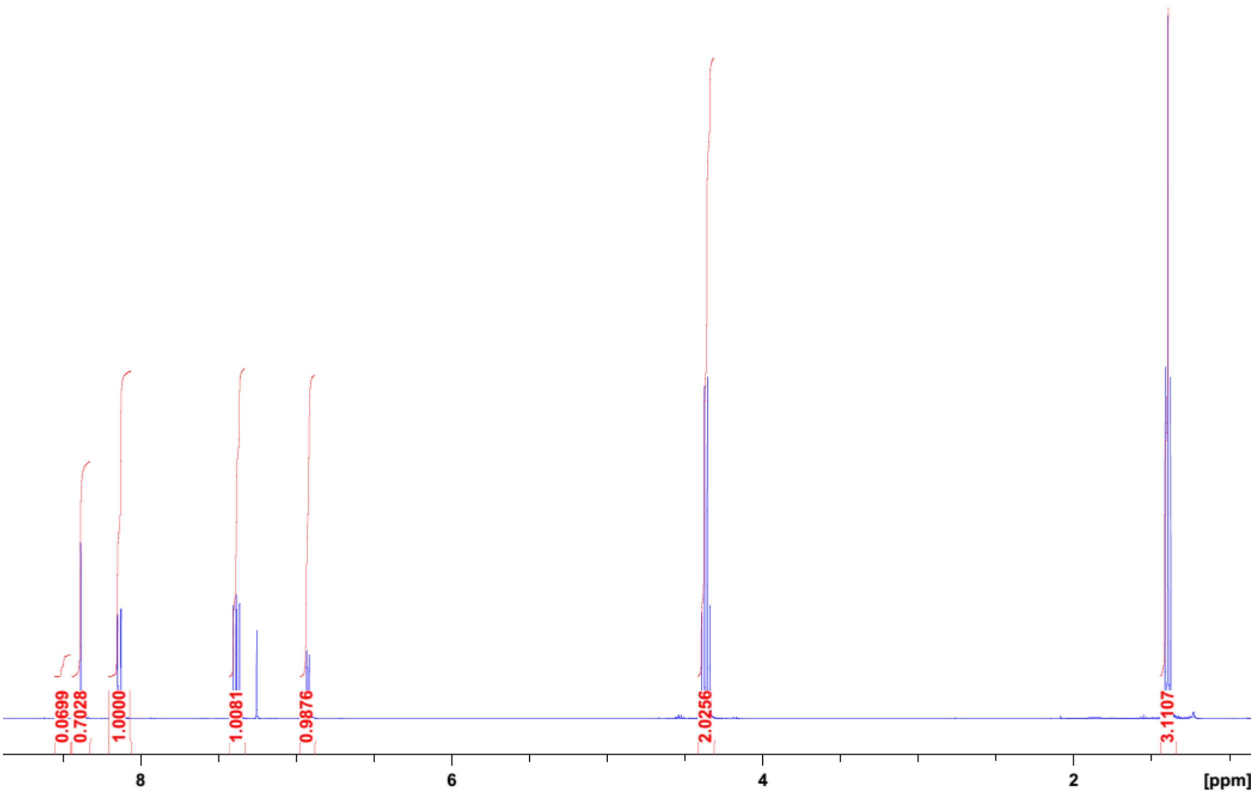

Figure S6. ^{1}H NMR of compounds **7,8**.

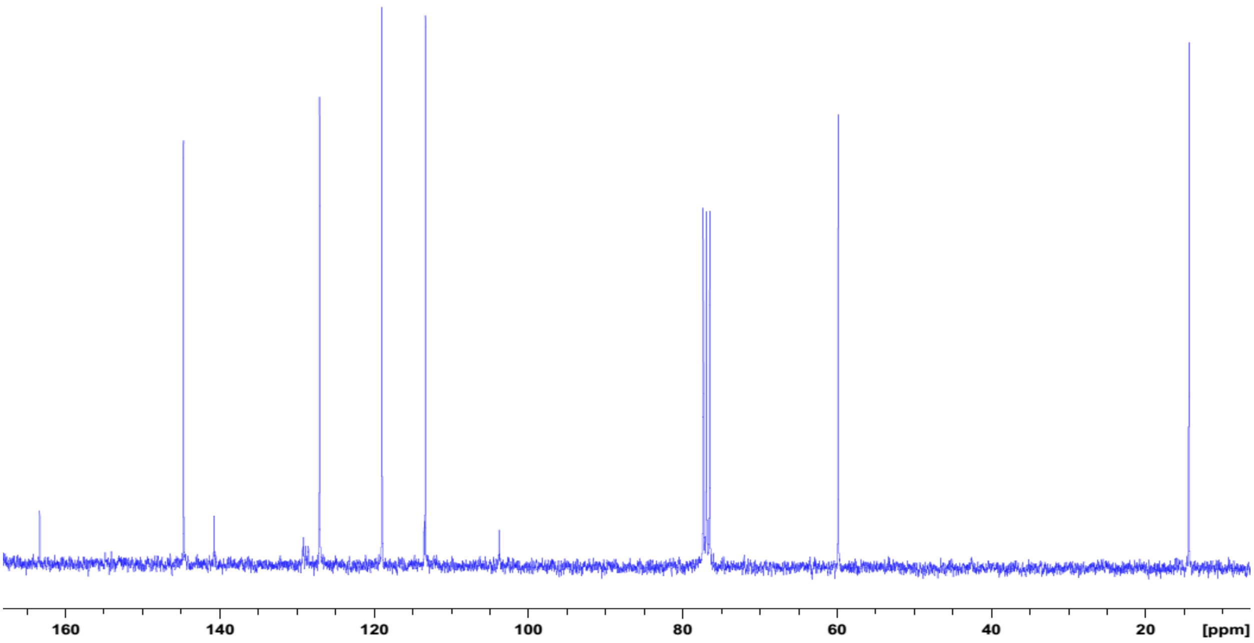

Figure S6. ^{1}H NMR of compounds **7,8**.

Dimethyl 5-methoxy-7D-pyrazolo[1,5-*a*]pyridine-2,3-dicarboxylate (6). [*cf.* Ref.[6]]

Was obtained according to the general procedure, but was held in water bath an hour instead of 5 min. ^{1}H-NMR ($CDCl_3$): 7.90 (d, 1H, J = 1.9), 6.83 (d, 1H, J =1.9), 3.99 (s, 3H), 3.89 (s, 3H) ppm.

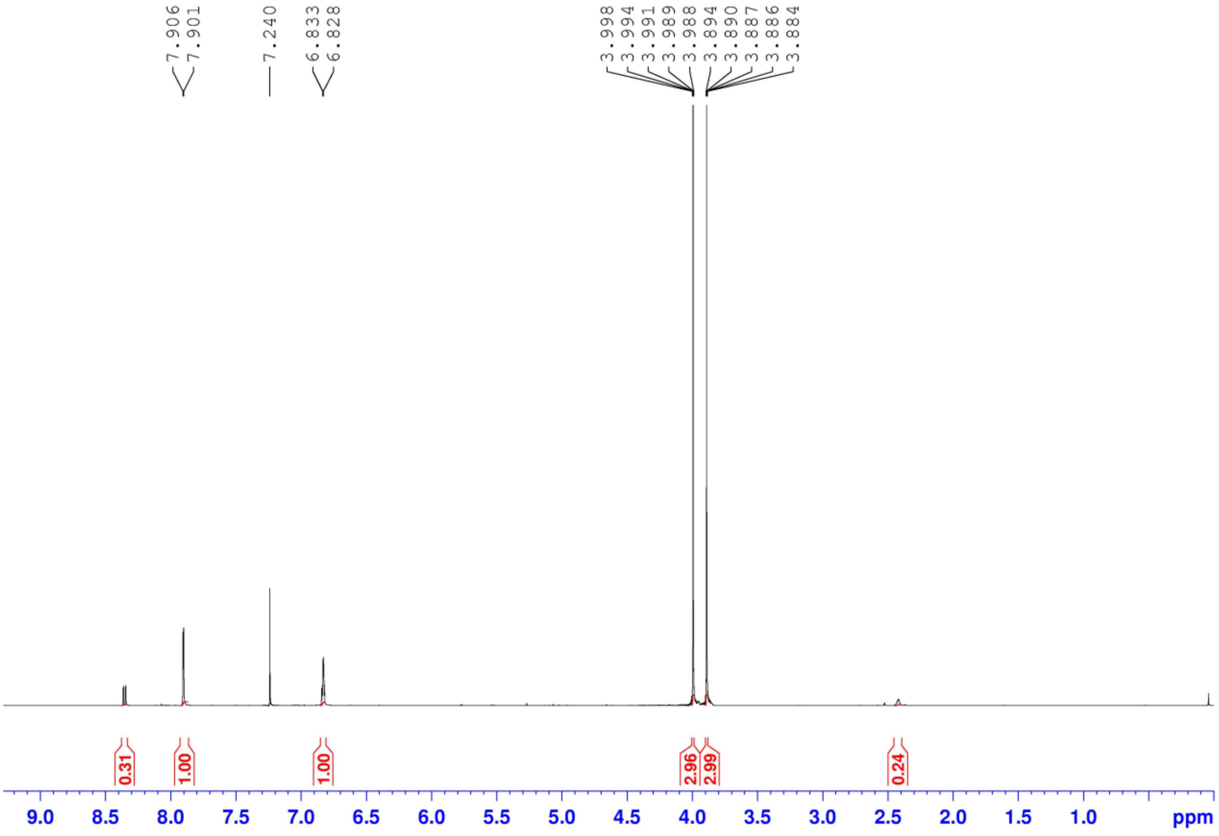

Figure S8. ^{1}H NMR of compound **6**.

Dimethyl 5-CD_3-7-D-pyrazolo[1,5-*a*]pyridine-2,3-dicarboxylate (5). [*cf.* Ref.[7]]

4-Methylpyridinium-*N*-amino mesitylenesulfonate (61 mg, 0.20 mmol) and K_2CO_3 (138 mg, 1.00 mmol) in D_2O (0.83 mol, 1.5 mL) were heated for 5 min in a water bath (80 °C). After cooling the mixture was evaporated. The residue was dissolved in D_2O (0.83 mol, 1.5 mL) and the solution was heated for 5 min in a water bath (80 °C). After cooling the mixture was evaporated. Acetylene (0.20 mmol) in acetonitrile (5 mL) was added to the residue. The mixture was stirred for 10 min and then chloranile (37 mg, 0.15 mmol) was added. After an hour the mixture was evaporated, the residue rinsed with chloroform and the resulting solution passed through a thin silica gel layer. Evaporation gave 17 mg (34%) of colourless crystals. ^{1}H-NMR ($CDCl_3$): 7.93 (d, 1H, J = 1.9 Hz), 6.85 (d, 1H, J = 1.9 Hz), 4.02 (s, 3H), 3.91 (s, 3H) ppm. ^{13}C NMR ($CDCl_3$): 163.5, 162.9, 147.6, 141.8, 139.6, 128.0 (t), 118.4, 117.7, 101.7, 53.0, 51.7 ppm. HRMS (EI): M^+, found 252.1042. $C_{12}H_8D_4N_2O_4$ requires 252.1043.

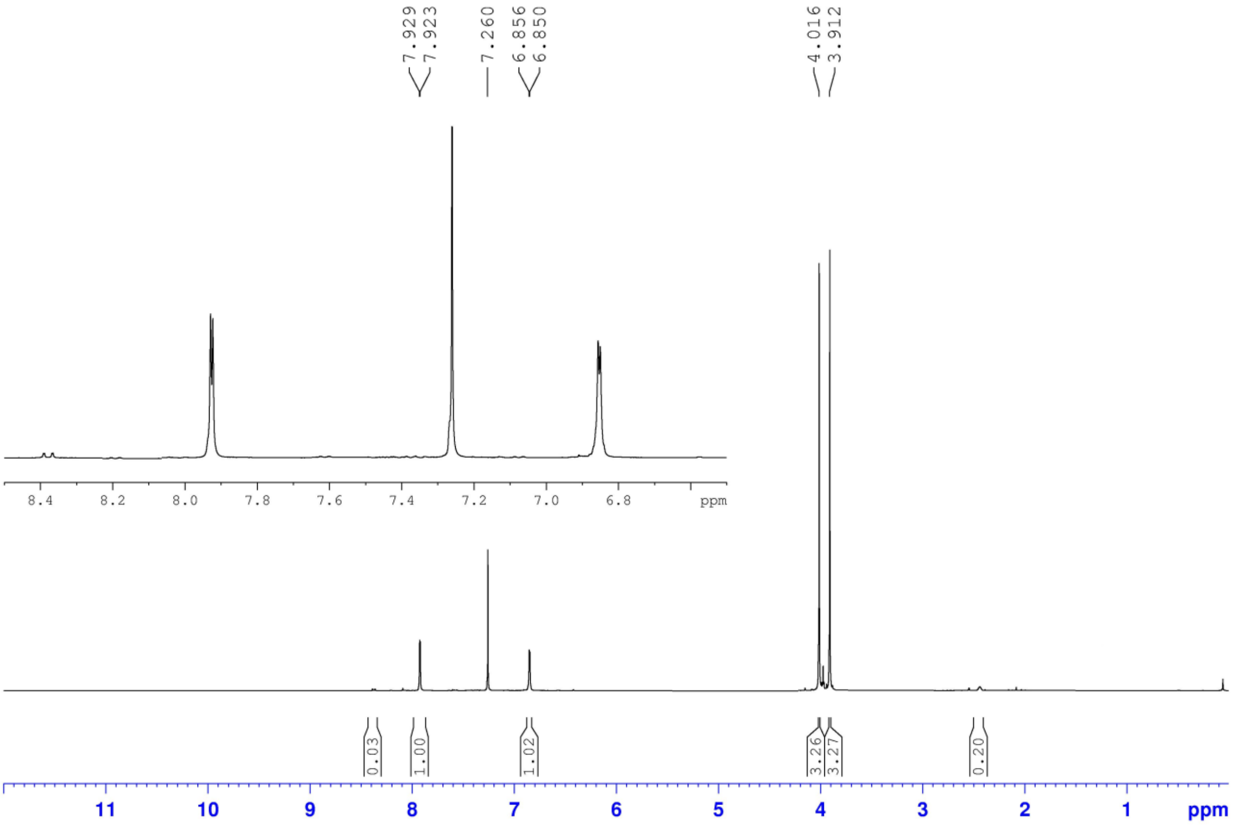

Figure S9. ^{1}H NMR of compound **5**.

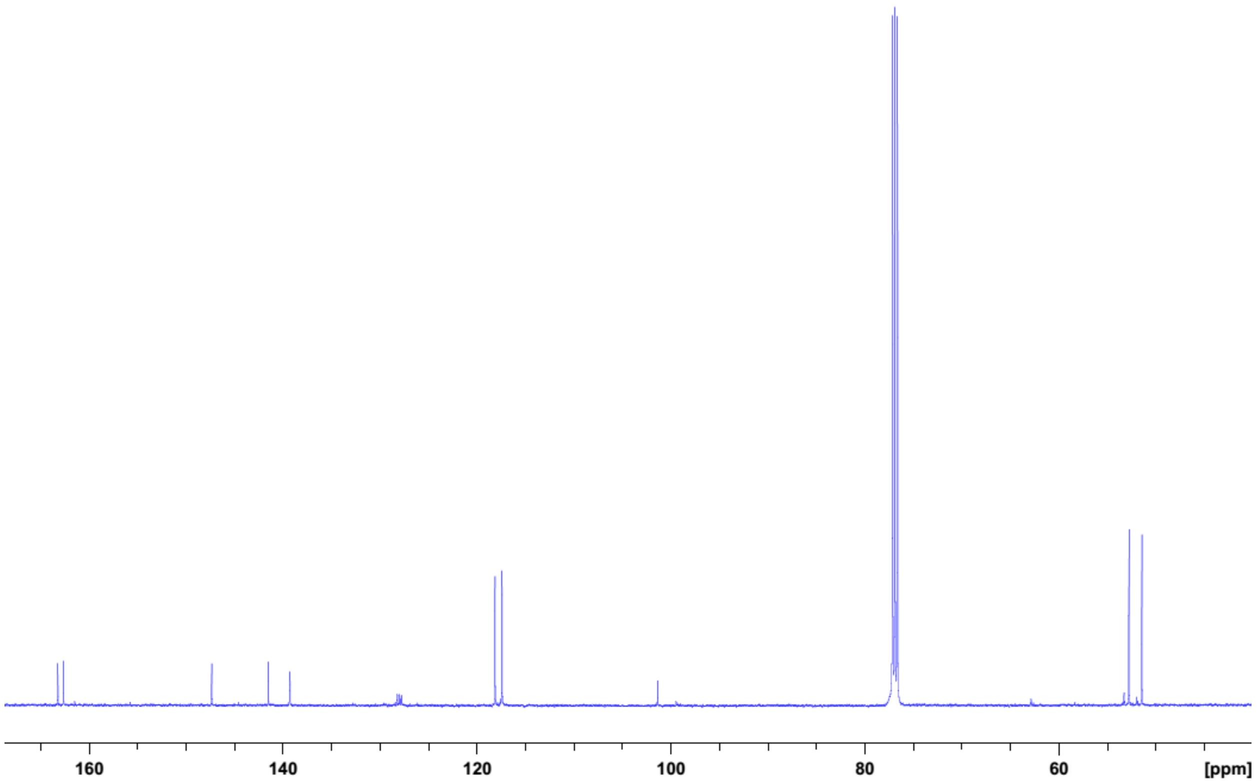

Figure S10. ^{13}C NMR of compound **5**.

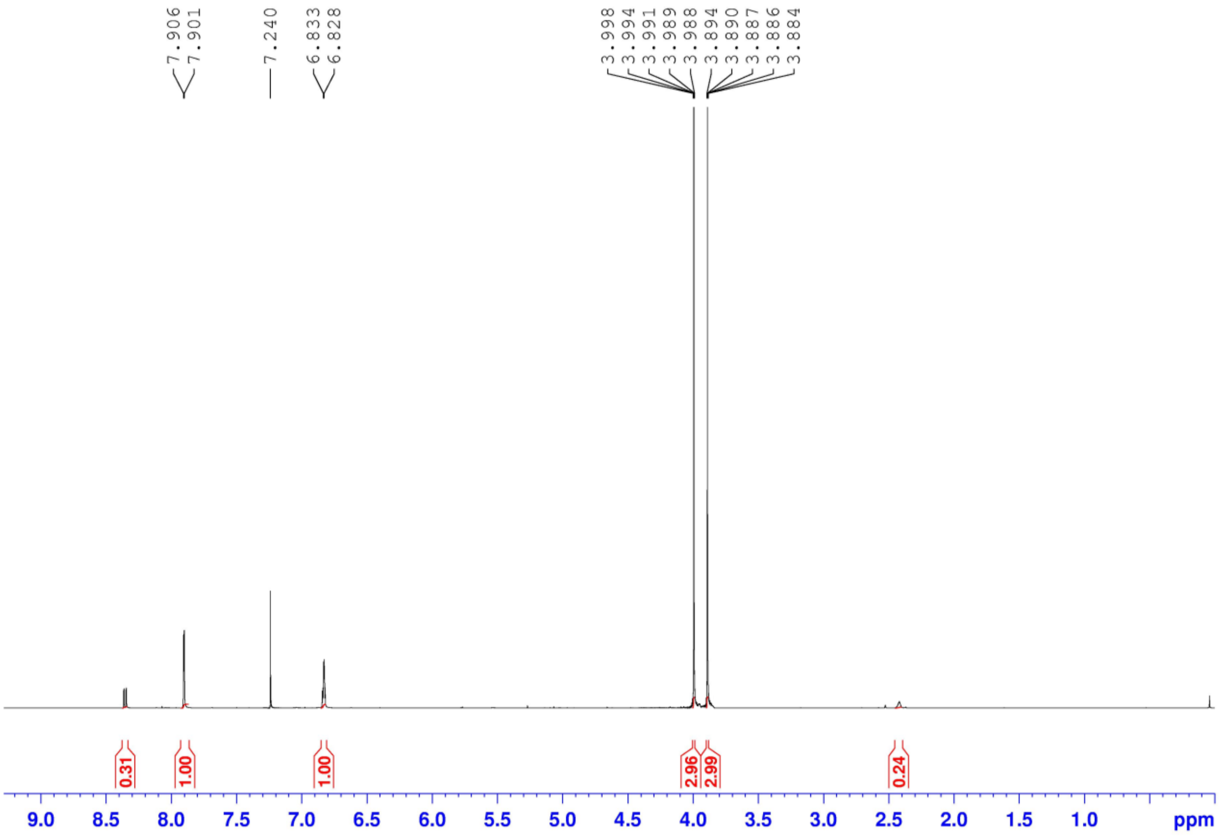

Figure S11. ^{1}H NMR of compound **5** after first deuteration step.

Synthesis of 7-deutero-1,2,4-triazolo[1,5-*a*]pyridines

General procedure for 7-D-1,2,4-triazolo[1,5-*a*]pyridines

To a basic D_2O solution prepared by mixing 1.5 mL D_2O with 336 mg (3 mmol) *t*-BuOK 36.4 mg (0.2 mmol) of 1-aminopyridinium tetrafluoroborate was added. The solution became violet in color. After that 0.2 mL MeCN-d_3, or 0.2 mmol of benzonitrile or 4-cyanopyridine was added in one portion without solvent. The mixture was vigorously stirred for overnight and then was extracted with CH_2Cl_2 (3 × 1 mL). Organic extracts were combined, dried over Na_2SO_4 and evaporated. Solid residue was chromatographed on Al_2O_3 to afford pure products.

2-CD$_3$-7-D-1,2,4-triazolo[1,5-*a*]pyridine (15). [*cf.* Ref.[8]]

^{1}H-NMR ($CDCl_3$): 7.60 (dd, 1H, J = 8.9 Hz, 1.3 Hz), 7.42 (dd, 1H, J = 8.9 Hz, 6.9 Hz), 6.91 (dd, 1H J = 6.9 Hz, 1.3Hz) ppm. ^{13}C NMR ($CDCl_3$): 163.8, 161.3, 129.2, 115.8, 112.9 (C^7 and CD_3 are not visible due to low intensity) ppm. HRMS (EI): M^+, found 137.0893. $C_{12}H_8D_4N_2O_4$ requires 137.0891.

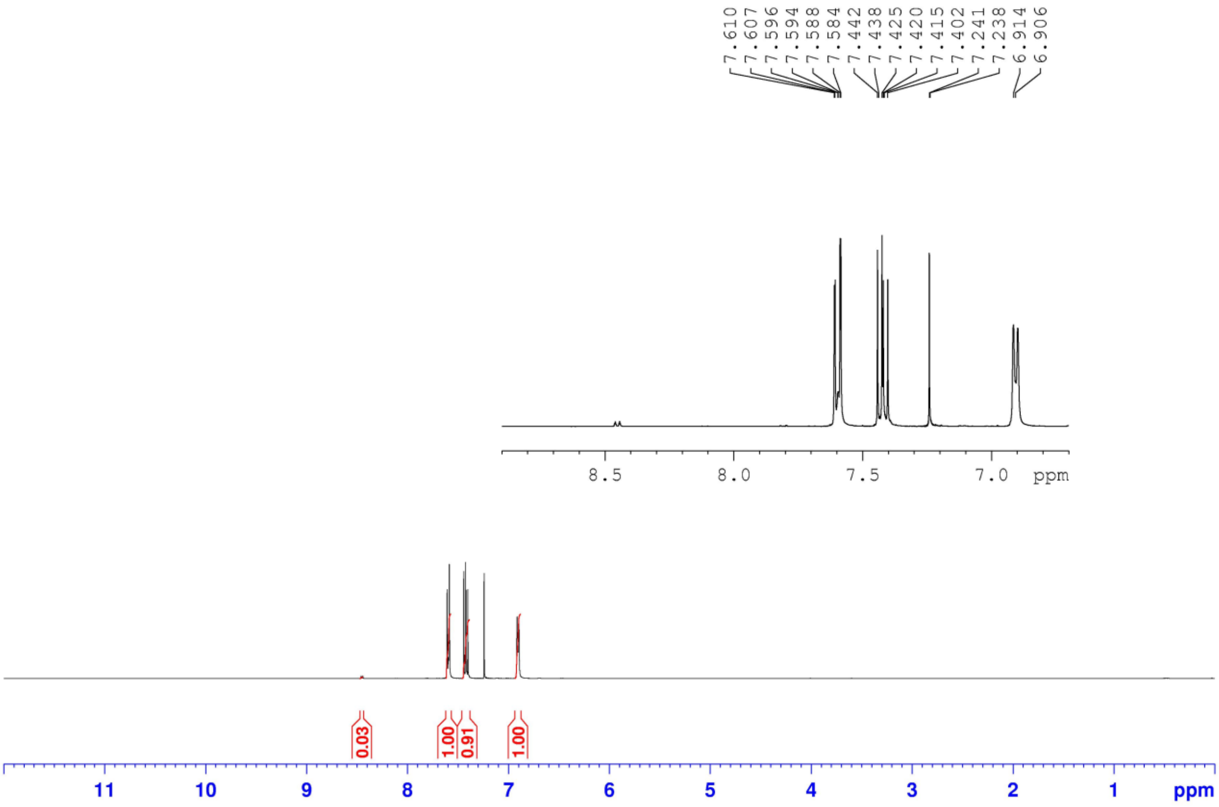

Figure S12. ^{1}H NMR of compound **15**

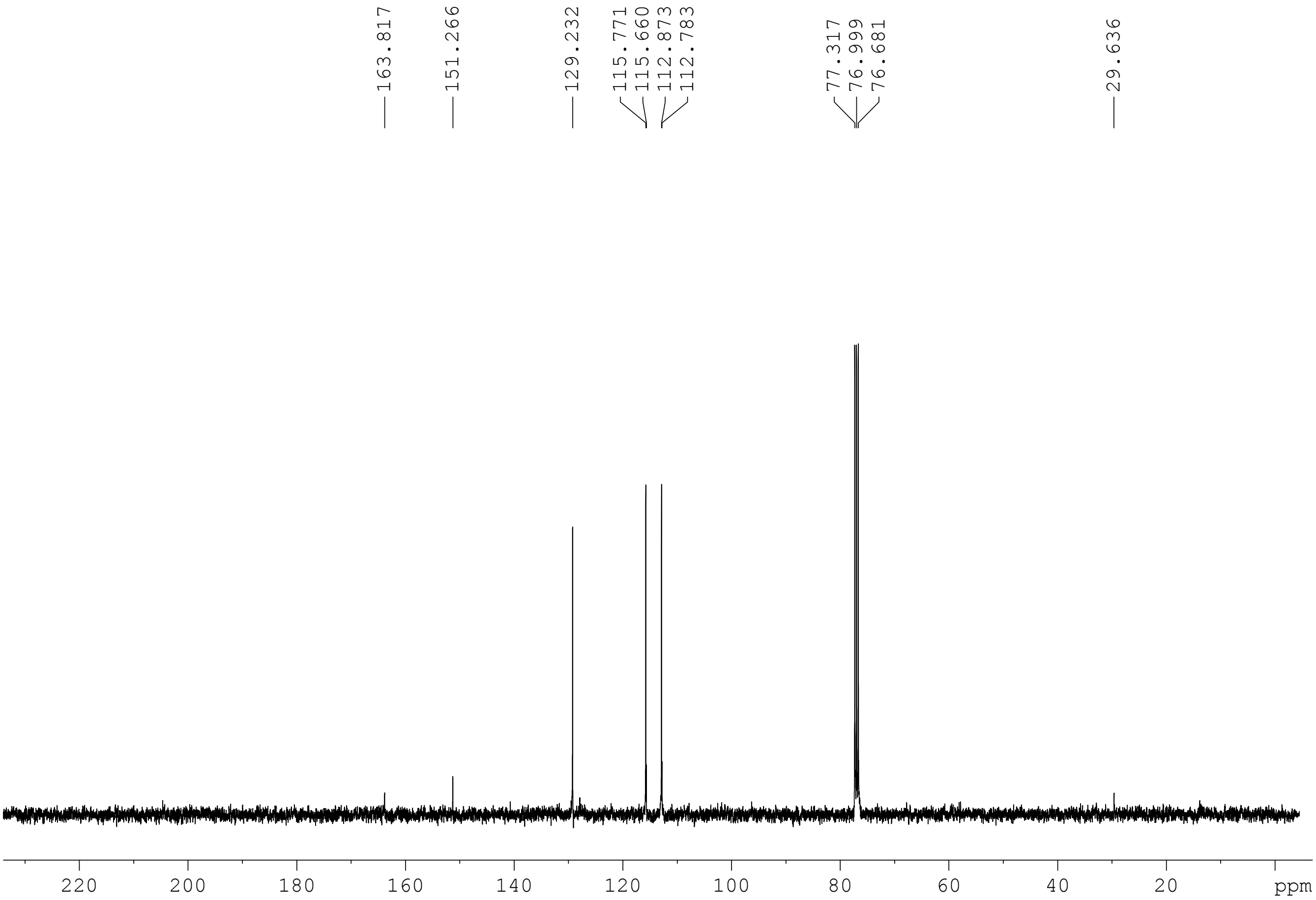

Figure S13. ^{13}C NMR of compound **15**

2-CH_3-7-D-1,2,4-triazolo[1,5-*a*]pyridine. (mixture of CH_3-, CH_2D- and CHD_2- products) (14).

^{1}H-NMR ($CDCl_3$): 7.60 (dd, 1H, J = 8.9 Hz, 1.3 Hz), 7.42 (dd, 1H, J = 8.9 Hz, 6.9 Hz), 6.91 (dd, 1H J = 6.9 Hz, 1.3Hz), 2.53 (s, CH_3), 2.52 (t, J = 2.3 Hz, CH_2D), 2.50 (pent, J = 2.2 Hz, CHD_2) ppm.

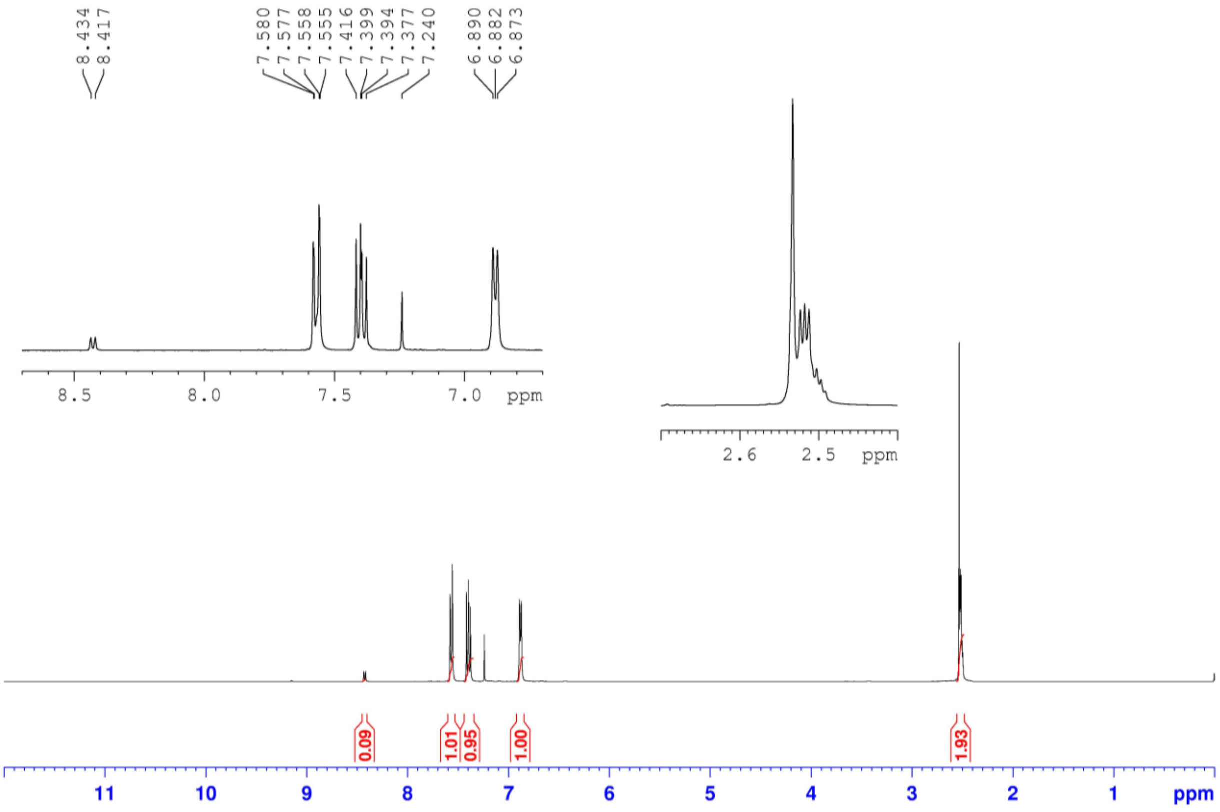

Figure S14. ^{1}H NMR of compound **14**

2-Ph-7-D-1,2,4-triazolo[1,5-*a*]pyridine (16). [*cf.* Ref.[9]]

^{1}H-NMR ($CDCl_3$): 8.26 (m, 2H), 7.71 (dd, 1H, J = 9.0 Hz, 1.2 Hz), 7.40-7.49 (m, 4H), 6.94 (dd, 1H, J = 6.9 Hz, 1.2 Hz) ppm. ^{13}C NMR ($CDCl_3$): 164.2, 151.7, 130.8, 130.0, 129.4, 128.6, 128.0 (t, J = 28.7 Hz), 127.3, 116.3, 113.4 ppm. HRMS (EI): M^+, found 196.0858. $C_{12}H_8D_4N_2O_4$ requires 196.0859.

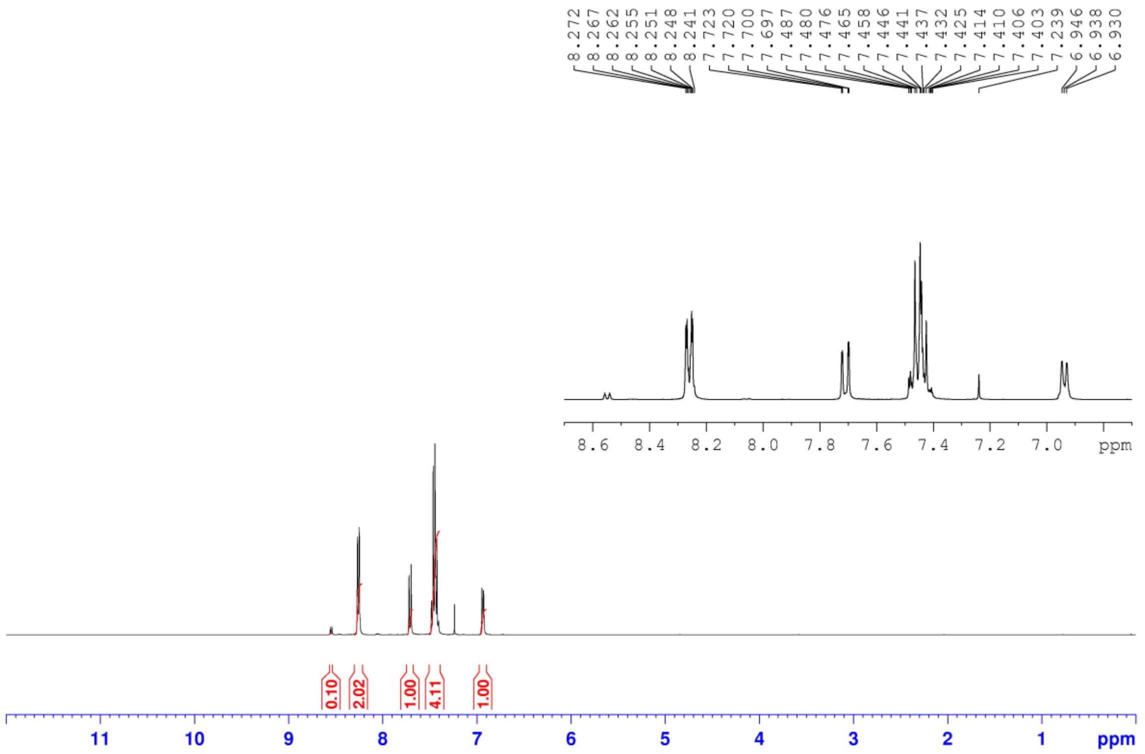

Figure S15. ^{1}H NMR of compound **16**

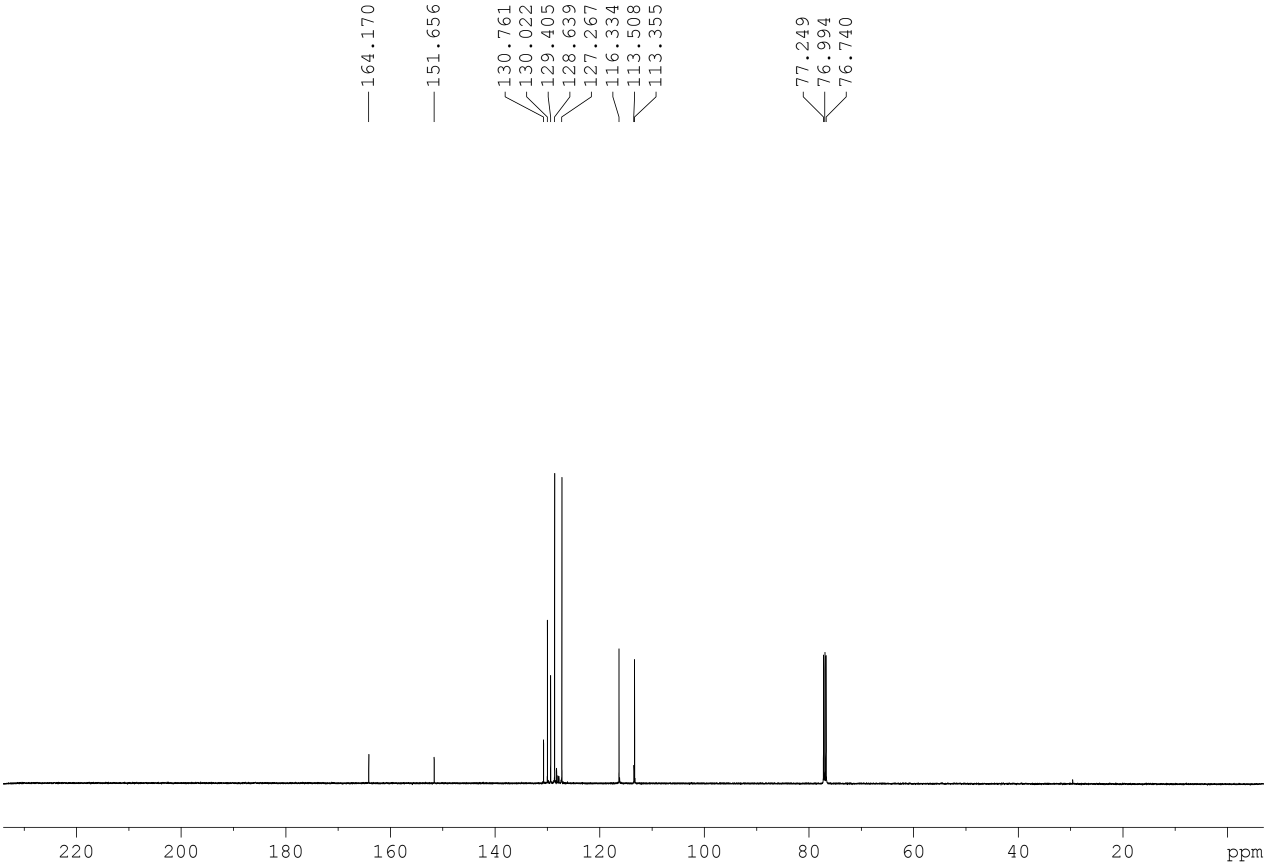

Figure S16. ^{13}C NMR of compound **16.**

2-(4-Pyridyl)-7-D-1,2,4-triazolo[1,5-*a*]pyridine (17). [*cf.* Ref.[9]]

^{1}H-NMR ($CDCl_3$): 8.69 (m, 2H), 8.06 (m, 2H), 7.71 (dd, 1H, J = 9.0 Hz, 1.2 Hz), 7.48 (dd, 1H, J = 9.0 Hz, 7.0 Hz), 6.99 (dd, 1H, J = 7.0 Hz, 1.2 Hz) ppm. ^{13}C NMR ($CDCl_3$): 161.8, 151.6, 150.3, 138.1, 129.9, 128.1 (t, J = 28.8 Hz), 121.2, 116.7, 114.1 ppm. HRMS (EI): M^+, found 197.0815. $C_{12}H_8D_4N_2O_4$ requires 196.0812.

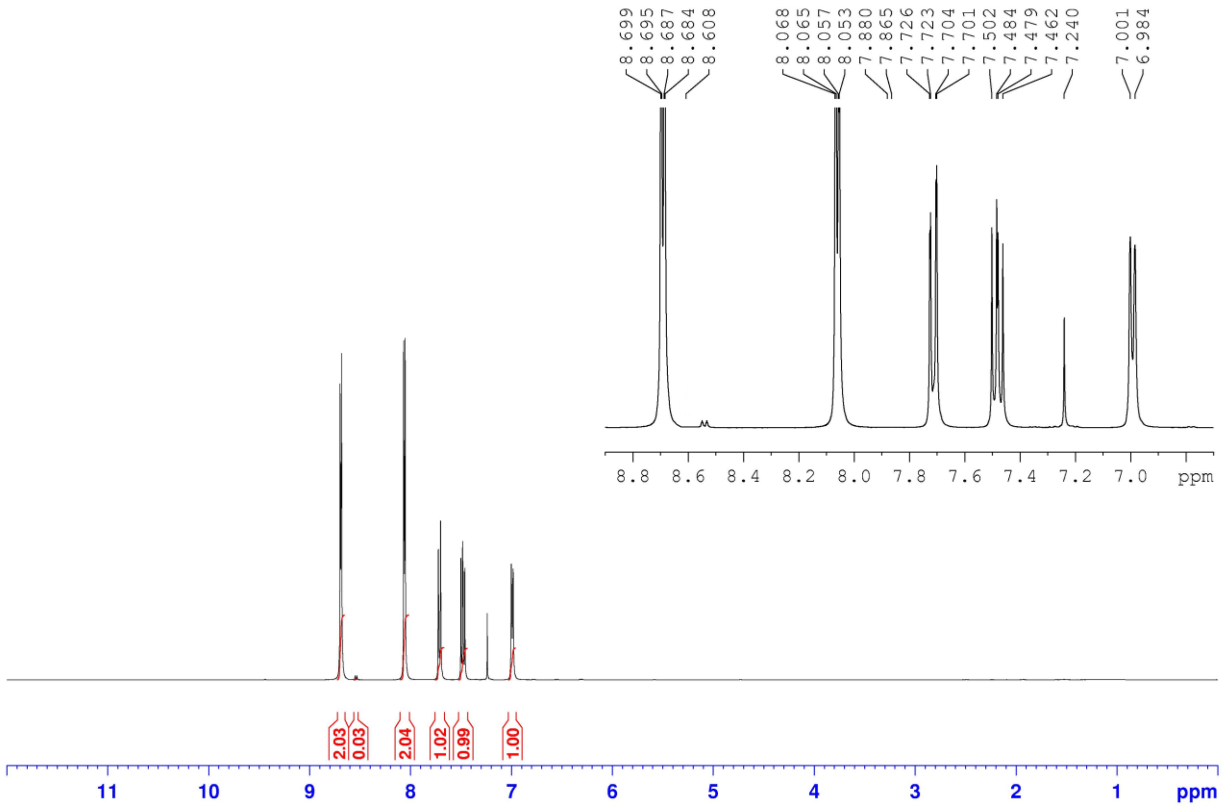

Figure S17. ^{1}H NMR of compound **17**.

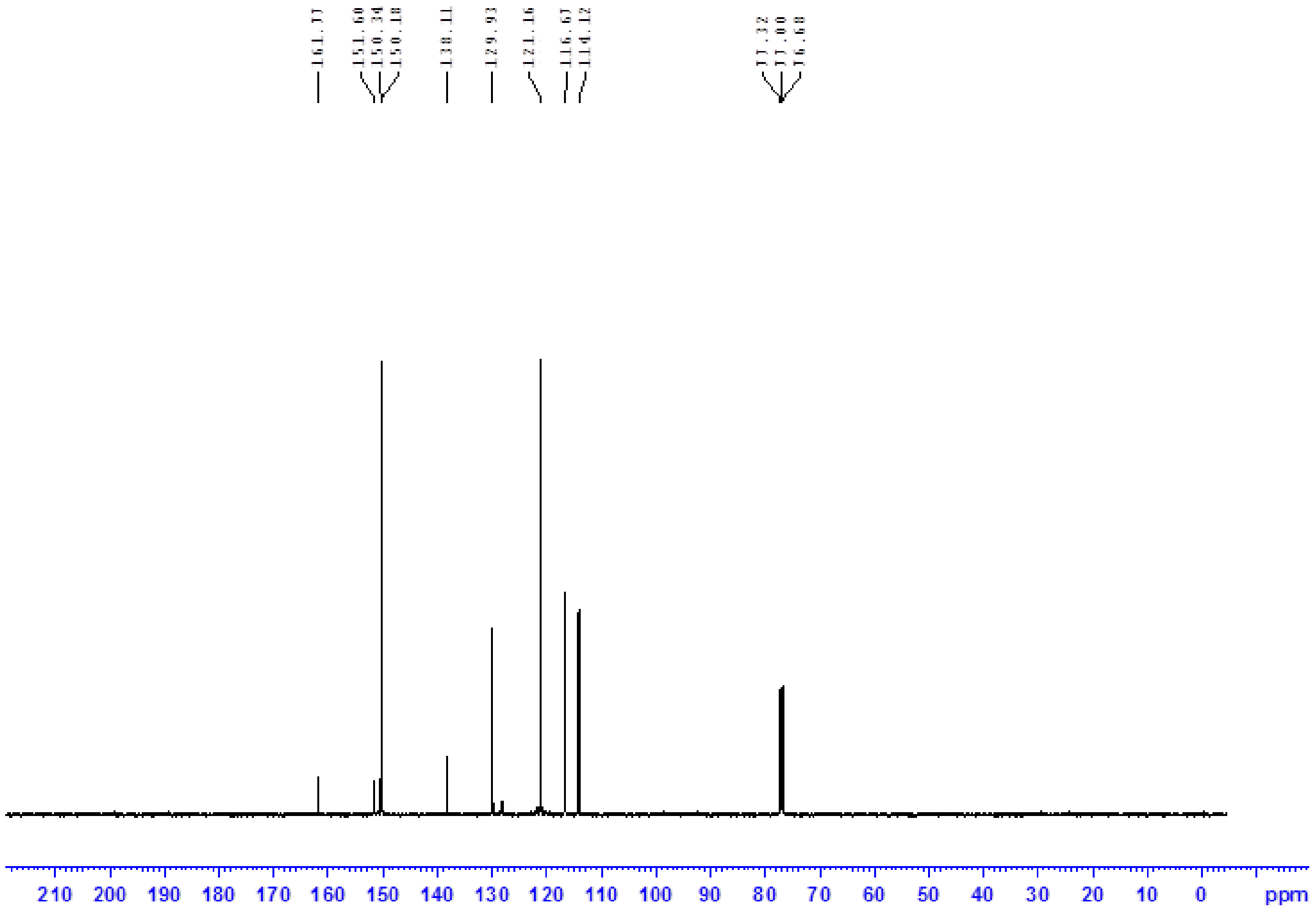

Figure S18. ^{1}H NMR of compound **17.**

Quantum chemistry studies

DFT quantum chemical calculations were performed with GAMESS[10] package at the M06-2X/6-31+G(d,p) level of theory with PCM SMD solvation. The nature of stationary points was confirmed with hessians calculation. For transition states one imaginary mode was observed. ZPVEs were used without correction. Thermodynamic properties and activation free energies for the reactions were calculated at 298.15 K and 1 atm for gas or 1 M solution.

N-aminopyridinium cations acidity calculations.

Simple thermodynamic cycle (Scheme 1) was applied to calculate pKa values[11].

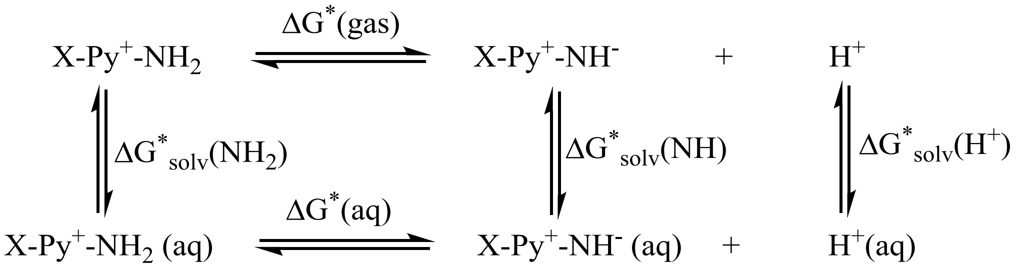

Scheme 1. Thermodynamic cycle.

From this cycle $\Delta G^*(\text{aq}) = \Delta G^*(\text{aq}) + \Delta\Delta G^*(\text{solv})$, where $\Delta\Delta G^*(\text{solv}) = \Delta G^*_{\text{solv}}(\text{NH}) + \Delta G^*_{\text{solv}}(\text{H}^+)$ - - $\Delta G^*_{\text{solv}}(\text{H}^+)$, and $\text{p}K_\text{a} = \Delta G^*(\text{aq})/(RT\ln 10)$. For H^+ we used following empirical values: $\Delta G^\text{o}_{\text{gas}}(\text{H}^+) = -26.3$ kJ mol^{-1} and $\Delta G^*_{\text{solv}}(\text{H}^+) = -1112.5$ kJ mol^{-1}

Different fucnctionals and basis sets were applied to calculate pK_a of 1-aminopyridinium cation (Figure S19). Two different values of the pK_a were found in literature 11.47[12] and 13.6[13]. Most of our calculations data are close to the first value. So we choose this value as reference.

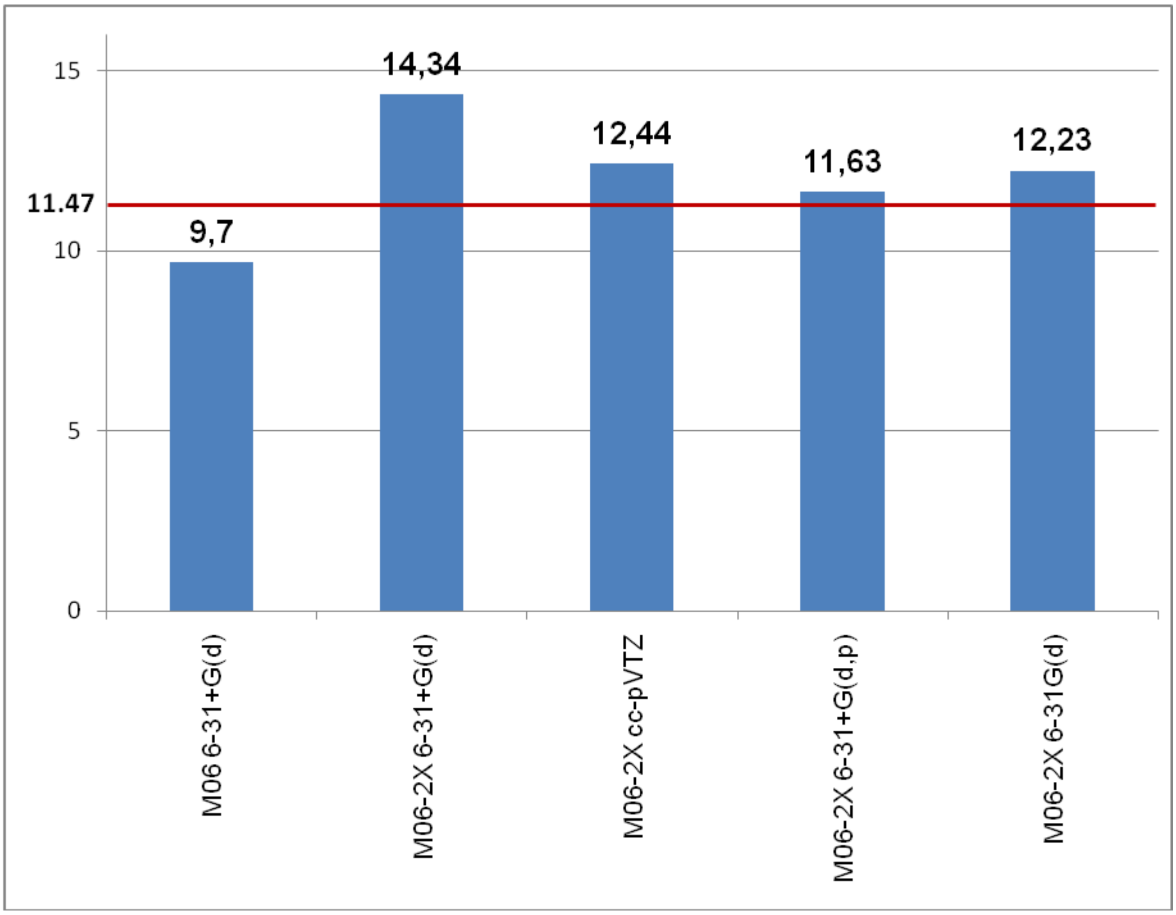

Figure S19. Calculated pKa of 1-aminopyridinium carion vs basis set.

Optimized structures of *N*-aminopyridinium cations, corresponded *N*-imines and C-ylides.

**1-aminopyridinium cation, *N*-imine and C-ylide**							
C	0.000000	0.000000	0.000000	C	0.000000	0.000000	0.000000
C	0.000000	0.000000	1.392944	C	0.000000	0.000000	1.397243
C	1.213548	0.000000	2.084914	C	1.242494	0.000000	2.023344
C	2.394845	-0.000068	1.370407	C	2.411484	0.000000	1.286940
N	2.365239	-0.000163	0.017946	N	2.405468	0.000000	-0.090116
C	1.208570	-0.000106	-0.674849	C	1.176397	0.000000	-0.714096
N	3.544190	-0.000236	-0.755034	N	3.471039	0.000000	-0.851302
H	1.320811	-0.000139	-1.752355	H	1.243167	0.000000	-1.793070
H	-0.921666	0.000113	-0.569399	H	-0.928266	0.000000	-0.560790
H	3.377610	0.000147	1.828284	H	3.395385	0.000000	1.738150
H	1.250378	-0.000110	3.167756	H	1.328578	0.000000	3.104603
H	4.084077	0.836808	-0.548326	H	4.277905	0.000000	-0.226041
H	4.084352	-0.836915	-0.547648	H	-0.921749	0.000000	1.964592
H	-0.937430	0.000143	1.939250				

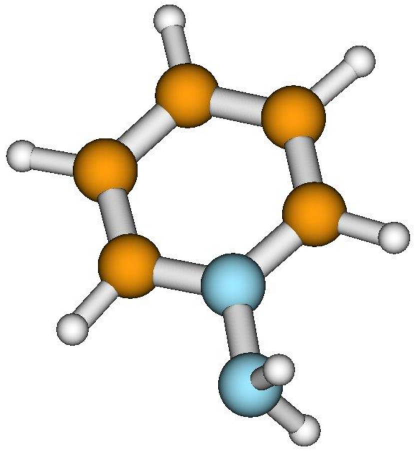

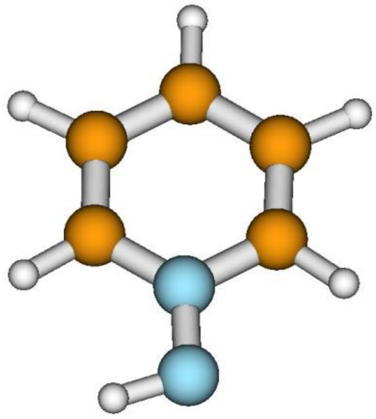

C	0.000000	0.000000	0.000000
C	0.000000	0.000000	1.381951
C	1.224936	0.000000	2.072578
C	2.377505	0.008417	1.330784
N	2.294933	0.055406	-0.026286
C	1.179206	0.042533	-0.796175
N	3.561922	0.082543	-0.677848
H	-0.949756	-0.020210	-0.526534
H	3.376922	-0.031915	1.746467
H	1.278796	-0.026585	3.154020
H	3.818923	1.065307	-0.768848
H	3.351042	-0.240924	-1.620893
H	-0.932587	-0.012286	1.939684

C	0.000000	0.000000	0.000000
C	0.000000	0.000000	1.418151
C	1.148543	0.000000	2.219297
C	2.389194	0.000202	1.610175
N	2.437323	0.000215	0.265856
C	1.300648	-0.000006	-0.498359
N	3.730077	0.000390	-0.311599
H	3.346553	0.000479	2.113916
H	1.100098	-0.000292	3.303917
H	3.807297	0.825844	-0.900628
H	3.807269	-0.824630	-0.901261
H	-0.956450	-0.000026	1.942391
H	1.532490	-0.000172	-1.564753

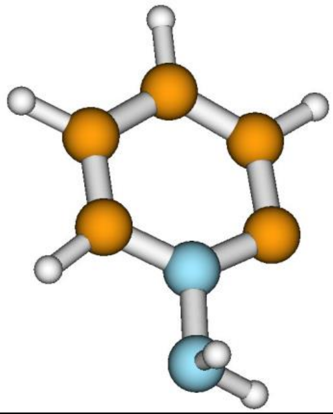

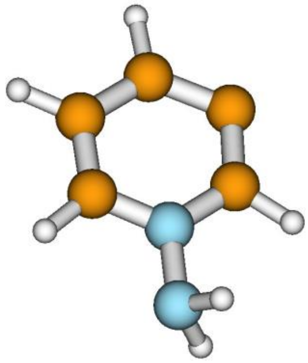

C	0.000000	0.000000	0.000000
C	0.000000	0.000000	1.430014
C	1.331893	0.000000	1.939172
C	2.469652	-0.000077	1.159759
N	2.357986	0.000010	-0.189916
C	1.134073	0.000112	-0.779544
N	3.549462	0.000163	-0.939790
H	3.491952	-0.000214	1.521857
H	1.499186	0.000037	3.014937
H	3.568355	0.830049	-1.525965
H	3.568042	-0.829076	-1.526902
H	1.143275	0.000249	-1.866383
H	-0.945120	-0.000052	-0.540864

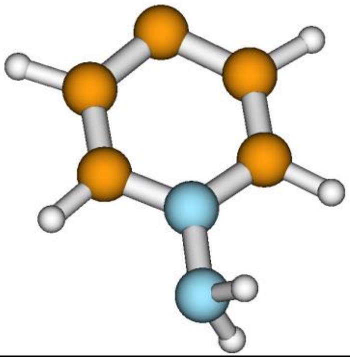

4-methyl-1-aminopyridinium cation, *N*-imine and C-ylide

C	0.000000	0.000000	0.000000
C	0.000000	0.000000	1.406733
C	1.240940	0.000000	2.054320
C	2.411338	-0.000658	1.320286
N	2.365520	-0.001244	-0.026637
C	1.187363	-0.000987	-0.694954
N	3.607065	-0.001073	-0.691597
H	-0.931846	0.001216	-0.555457
H	3.408272	-0.000203	1.744332
H	1.305146	0.001227	3.136583
H	3.684260	0.834777	-1.265760
H	3.685316	-0.837907	-1.264193
C	-1.288330	-0.006279	2.168496
H	-1.121944	0.074506	3.242586
H	-1.924564	0.822756	1.845872
H	-1.834111	-0.934027	1.968948
H	1.250968	-0.000830	-1.777305

C	0.000000	0.000000	0.000000
C	0.000000	0.000000	1.402342
C	1.258630	0.000000	1.997163
C	2.421608	-0.000804	1.246949
N	2.402745	-0.001658	-0.125522
C	1.164901	-0.000740	-0.729693
N	3.459172	-0.003186	-0.906167
H	-0.936371	0.000570	-0.550635
H	3.408680	-0.000611	1.691511
H	1.359673	0.000432	3.078523
H	4.274421	-0.004047	-0.291846
C	-1.277695	-0.001966	2.196519
H	1.213929	-0.000545	-1.809818
H	-1.070647	0.016603	3.269139
H	-1.895130	0.870269	1.958457
H	-1.874812	-0.895219	1.985927

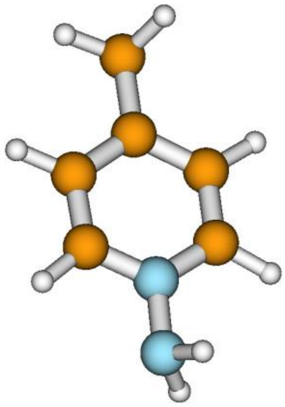

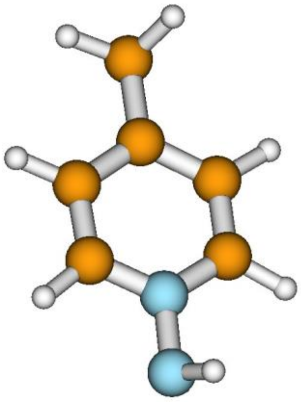

C	0.000000	0.000000	0.000000
C	0.000000	0.000000	1.385928
C	1.249648	0.000000	2.043906
C	2.390990	0.007997	1.289154
N	2.294048	0.050511	-0.066987
C	1.163692	0.037907	-0.814268
N	3.553063	0.075675	-0.735462
H	-0.958558	-0.018866	-0.514027
H	3.394804	-0.028618	1.694622
H	1.320594	-0.025472	3.126156
H	3.776877	1.059037	-0.887176
H	3.342642	-0.305462	-1.656674

C	0.000000	0.000000	0.000000
C	0.000000	0.000000	1.464348
C	1.344250	0.000000	2.045423
C	2.451907	0.005856	1.276913
N	2.381892	0.015436	-0.104128
C	1.143424	0.005894	-0.714753
N	3.518185	-0.016212	-0.912905
H	-0.945945	-0.004713	-0.530128
H	3.456197	0.006321	1.687706
H	1.456468	-0.004133	3.124267
H	4.095181	0.801994	-0.739799
H	4.055766	-0.859730	-0.732297

C	-1.270853	-0.012401	2.190523
H	-1.304048	-0.885895	2.849145
H	-1.335967	0.878229	2.823553
H	-2.147247	-0.037738	1.540194

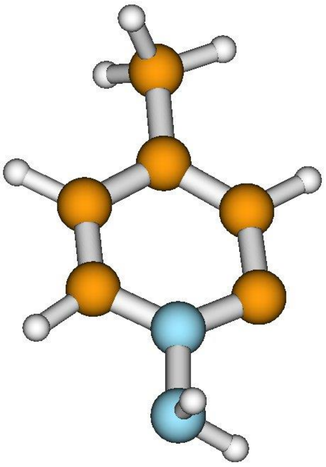

C	-1.125302	-0.000899	2.210948
H	1.183541	0.005235	-1.796968
H	-1.080003	-0.001187	3.294548
H	-2.105831	-0.001277	1.747629

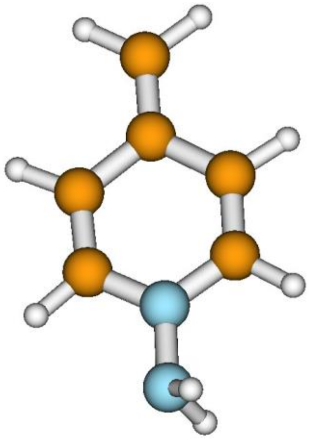

4-methoxy-1-aminopyridinium cation

C	0.000000	0.000000	0.000000
C	0.000000	0.000000	1.415022
C	1.245079	0.000000	2.089549
C	2.406414	0.000856	1.351571
N	2.375684	-0.000445	-0.006799
C	1.190557	-0.001979	-0.679928
N	3.549255	-0.001738	-0.795850
H	-0.939887	-0.001216	-0.538166
H	3.389103	0.003266	1.807891
H	1.314367	-0.000045	3.168886
H	4.083262	0.847417	-0.632248
O	-1.180173	-0.000584	2.002489
H	1.277391	-0.005215	-1.758565
C	-1.297173	-0.010795	3.443988
H	-2.367630	-0.011387	3.639337
H	-0.841336	-0.916976	3.852968
H	-0.839899	0.887717	3.867959
H	4.104525	-0.829370	-0.597288

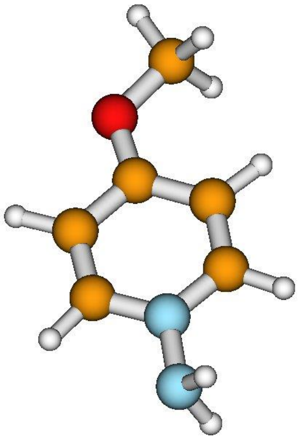

C	0.000000	0.000000	0.000000
C	0.000000	0.000000	1.405521
C	1.242416	0.000000	2.040555
C	2.412295	0.000063	1.290081
N	2.412928	0.000063	-0.084863
C	1.177365	0.000009	-0.706855
N	3.490673	0.000159	-0.869044
H	-0.941207	-0.000015	-0.537633
H	3.391309	0.000134	1.751421
H	1.340808	-0.000039	3.118679
H	4.299713	0.000181	-0.244542
O	-1.216148	0.000037	2.024575
H	1.235779	0.000011	-1.786085
C	-1.242956	0.000608	3.445182
H	-2.296938	0.000945	3.725598
H	-0.757822	-0.895826	3.853441
H	-0.757427	0.897204	3.852721

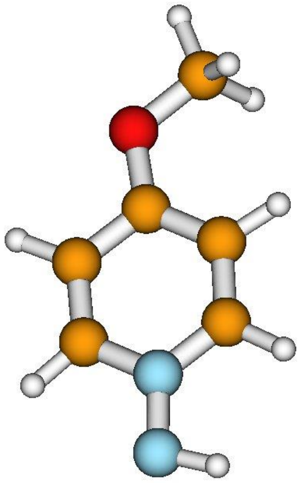

C	0.000000	0.000000	0.000000
C	0.000000	0.000000	1.397157
C	1.235522	0.000000	2.084308

C	2.385857	0.012493	1.330050
N	2.307436	0.059580	-0.023789
C	1.168822	0.041905	-0.784393
N	3.577887	0.093738	-0.691647
H	-0.963422	-0.024507	-0.501139
H	3.379924	-0.024797	1.758795
H	1.310529	-0.030237	3.162431
H	3.800165	1.077350	-0.848095
H	3.376552	-0.296383	-1.611574
O	-1.202557	-0.012641	2.021085
C	-1.253553	-0.020134	3.445317
H	-2.313178	-0.019363	3.702958
H	-0.780611	-0.920746	3.855770
H	-0.775976	0.873340	3.865569

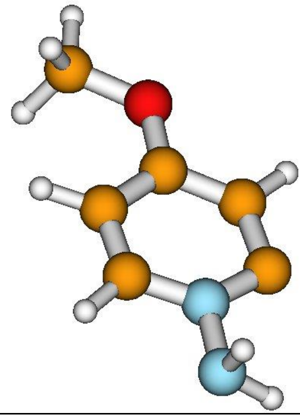

Optimized structures of dihydro ethyl pyrazolo[1,5-*a*]pyridine-3-carboxylate intermediates.

N	-1.999153	0.870962	0.451942
C	-1.124491	-0.336816	0.576012
C	-1.627562	-1.466126	-0.299048
C	-2.932701	-1.508609	-0.639357
C	-3.844091	-0.421479	-0.308563
C	-3.332492	0.765126	0.113132
C	0.224194	0.256500	0.188183
H	-0.913031	-2.217493	-0.621160
H	-3.314528	-2.335805	-1.232974
H	-4.911592	-0.526235	-0.467363
H	-3.921820	1.672850	0.224785
N	-1.239663	1.997626	-0.016622
C	0.061278	1.548433	-0.176948
H	0.814362	2.240066	-0.531923
C	1.469529	-0.499917	0.240035
O	2.555153	0.249012	-0.091646
O	1.542179	-1.682241	0.545933
C	3.830003	-0.436757	-0.082130
C	4.898598	0.578866	-0.445315
H	3.789636	-1.262620	-0.799745
H	3.992333	-0.864608	0.912313
H	-1.651103	2.445894	-0.835496
H	-1.123768	-0.648316	1.633766
H	5.880911	0.093883	-0.449684
H	4.719613	1.000240	-1.439846
H	4.923876	1.399038	0.279270

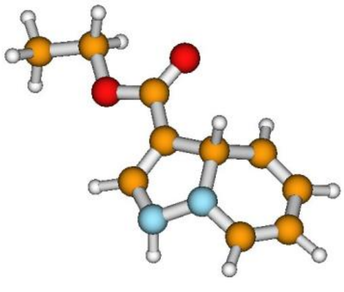

N	-2.057062	0.895753	-0.487616
C	-1.145390	-0.285841	-0.708027
C	-1.604762	-1.524995	0.031108
C	-2.820813	-1.603877	0.598828
C	-3.715226	-0.453678	0.629558
C	-3.267921	0.742827	0.193247
C	0.199220	0.281413	-0.257133
H	-0.894311	-2.344681	0.062022
H	-3.129322	-2.519035	1.098014
H	-4.708600	-0.535610	1.056226
H	-3.835760	1.662076	0.304513
N	-1.264924	2.025321	-0.044308
C	0.050530	1.592751	0.026135
H	0.821163	2.305493	0.292666
C	1.439182	-0.486595	-0.256067
O	2.509361	0.240026	0.163027
O	1.524808	-1.653550	-0.614396
C	3.783705	-0.447205	0.173345
C	4.843188	0.560844	0.581993
H	3.723985	-1.284230	0.876974
H	3.970539	-0.859665	-0.823171
H	-1.412496	2.840217	-0.639383
H	-1.103816	-0.510107	-1.788460
H	5.825284	0.075702	0.600502
H	4.885703	1.394378	-0.126697
H	4.640191	0.963612	1.579629

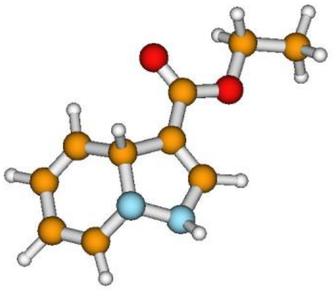

N	-1.626727	-0.699394	0.460075
C	-1.229554	-0.350059	-0.921745
C	-1.276313	1.145436	-1.098927
C	-2.125840	1.870122	-0.333945
C	-2.867840	1.279861	0.766883
C	-2.532371	0.025255	1.182093
C	0.124125	-1.119402	-1.052239
H	-0.671577	1.596420	-1.879383
H	-2.220304	2.939951	-0.504826
H	-3.595520	1.858607	1.322175
H	-2.892347	-0.446954	2.089819
N	-0.975857	-1.801238	0.929839
C	-0.029701	-2.097067	0.103149
H	0.632294	-2.927279	0.316293
C	1.363218	-0.218653	-0.961878
O	1.813602	-0.083794	0.295417

N	-1.942823	0.804276	0.207214
C	-1.066014	-0.395739	0.226677
C	-1.750209	-1.529111	-0.493436
C	-3.101684	-1.564941	-0.539913
C	-3.915228	-0.460137	-0.059157
C	-3.309437	0.728555	0.217355
C	0.238159	0.171778	-0.374398
H	-1.137659	-2.324764	-0.908719
H	-3.603397	-2.413430	-0.999151
H	-4.995113	-0.538004	-0.025504
H	-3.829312	1.662287	0.403825
N	-1.252190	1.962448	0.019873
C	-0.027100	1.669983	-0.260096
H	0.692375	2.451265	-0.471129
C	1.493602	-0.265140	0.368706
O	2.591224	-0.013349	-0.372292

O	1.861050	0.323587	-1.929107
C	2.947092	0.804573	0.493678
C	3.262607	0.815996	1.977345
H	2.674538	1.796275	0.119810
H	3.784549	0.435068	-0.106365
H	-1.943104	-0.850633	-1.606362
H	0.183422	-1.606038	-2.027877
H	4.112680	1.481002	2.165181
H	2.407427	1.176563	2.557074
H	3.524386	-0.185864	2.331773

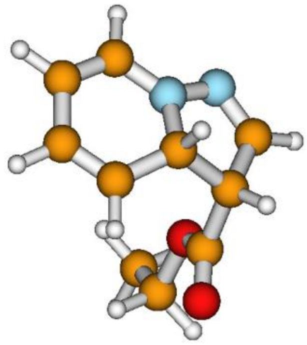

O	1.521677	-0.757730	1.477860
C	3.871950	-0.338830	0.235741
C	4.957212	0.016017	-0.763184
H	3.872868	-1.404044	0.486893
H	3.964096	0.228051	1.167397
H	-0.865111	-0.655818	1.281027
H	0.365839	-0.091432	-1.436659
H	5.937682	-0.225110	-0.338310
H	4.837851	-0.549942	-1.692626
H	4.939754	1.084145	-1.002300

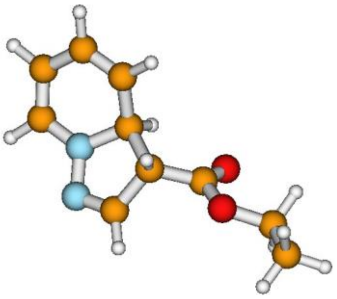

N	-1.949534	-0.865136	-0.015741
C	-1.065913	0.214126	-0.035365
C	-1.664133	1.513914	-0.019688
C	-3.031676	1.625495	0.049616
C	-3.877824	0.475387	0.104895
C	-3.295962	-0.768994	0.069942
C	0.231997	-0.263587	-0.042790
H	-1.002623	2.370617	-0.058701
H	-3.483862	2.613259	0.071992
H	-4.954246	0.566218	0.181060
H	-3.835079	-1.708632	0.106891
N	-1.261990	-2.110028	-0.171322
C	0.161070	-1.770307	0.147428
H	0.819801	-2.344485	-0.508907
C	1.410100	0.555609	-0.052045
O	2.551239	-0.204085	0.030891
O	1.446342	1.784400	-0.122402
C	3.797796	0.521448	0.039920
C	4.922125	-0.500221	0.069709
H	3.848407	1.157488	-0.849959
H	3.822337	1.177471	0.917166
H	-1.319016	-2.322075	-1.171033
H	0.373463	-2.071985	1.183317
H	5.890278	0.012907	0.082454
H	4.855683	-1.131637	0.961815
H	4.886673	-1.147007	-0.813191

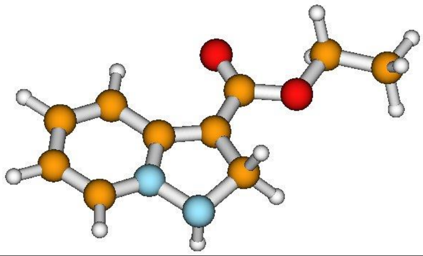

References

1. Y. Tamura, J. Minamikawa, M. Ikeda, *Synthesis.* **1977**, 1.

2. Lämsä M., Huuskonen J., Rissanen K., Pursiainen J. *Chem. Eur. J.*, **1998**, *4*, 84-92.

3. V. I. Supranovich, A. Yu. Vorob'ev, G. I. Borodkin, Y. V. Gatilov, V. G. Shubin, *Tetrahedron Lett.*, **2016**, *57*, 1093.

4. D. C. Mohan, C. Ravi, S. N. Raoa, S. Adimurthy, *Org. Biomol. Chem.*, **2015**, *13*, 3556.

5. Almirall S.A., Bach T. J., Perez Crespo D.; Llera Soldevila O., Esteve Trias C., Taboada Martinez L., patent, WO2015/86693 A1 (2015)

6. D. Möller, R. C. Kling, M. Skultety, K. Leuner, H. Hübner, P. Gmeiner, *J. Med. Chem.*, **2014**, *57*, 4861.

7. T. Sasaki, K. Kanematsu, A. Kakehi, *J. Org. Chem.*, **1971**, *36*, 2978.

8. T. Koike, T. Takai, Y. Hoashi, Patent EP2141150 A1, 2010.

9. S. Ueda, H. Nagasawa, *J. Am. Chem. Soc.*, **2009**, *131*, 15080.

10. M.W. Schmidt, K.K.Baldridge, J.A.Boatz, S.T.Elbert, M.S.Gordon, J.H.Jensen, S.Koseki, N.Matsunaga, K.A.Nguyen, S.Su, T.L.Windus, M.Dupuis, J.A.Montgomery, *J. Comput. Chem.*, **1993**, *14*, 1347.

11. G. C. Shields, P. G. Seybold. Computational approaches for the prediction of pKa values; J. Devillers, Ed.; CRC Press,Taylor & Francis Group, 2015.

12. T. Okamoto, M. Hirobe, Y. Tamai, and E. Yabe, *Chem. Pharm. Bull.* **1966**, *14*, 506.

13. J. Epsztajn, E. Lunt, and A. R. Katritzky, *Tetrahedron*, **1970**, *26*, 1665.
